# Distribution of vasotocin- and vasoactive intestinal peptide-like immunoreactivity in the brain of blue tit (*Cyanistes coeruleus*)

**DOI:** 10.3389/fnana.2015.00090

**Published:** 2015-07-14

**Authors:** Catherine M. Montagnese, Tamás Székely, András Csillag, Gergely Zachar

**Affiliations:** ^1^Department of Anatomy, Histology and Embryology, Semmelweis UniversityBudapest, Hungary; ^2^Department of Biology and Biochemistry, University of BathBath, UK

**Keywords:** neuropeptides, mapping, songbird, Paridae, avian brain

## Abstract

Blue tits (*Cyanistes coeruleus*) are songbirds, used as model animals in numerous studies covering a wide field of research. Nevertheless, the distribution of neuropeptides in the brain of this avian species remains largely unknown. Here we present some of the first results on distribution of Vasotocine (AVT) and Vasoactive intestinal peptide (VIP) in the brain of males and females of this songbird species, using immunohistochemistry mapping. The bulk of AVT-like cells are found in the hypothalamic supraoptic, paraventricular and suprachiasmatic nuclei, bed nucleus of the stria terminalis, and along the lateral forebrain bundle. Most AVT-like fibers course toward the median eminence, some reaching the arcopallium, and lateral septum. Further terminal fields occur in the dorsal thalamus, ventral tegmental area and pretectal area. Most VIP-like cells are in the lateral septal organ and arcuate nucleus. VIP-like fibers are distributed extensively in the hypothalamus, preoptic area, lateral septum, diagonal band of Broca. They are also found in the bed nucleus of the stria terminalis, amygdaloid nucleus of taenia, robust nucleus of the arcopallium, caudo-ventral hyperpallium, nucleus accumbens and the brainstem. Taken together, these results suggest that both AVT and VIP immunoreactive structures show similar distribution to other avian species, emphasizing evolutionary conservatism in the history of vertebrates. The current study may enable future investigation into the localization of AVT and VIP, in relation to behavioral and ecological traits in the brain of tit species.

## Introduction

Passerine birds (songbirds) are one of the major vertebrate groups of organisms for investigating ecology, behavior and evolution (Bennett and Owens, 2002; Grant and Grant, 2014). Songbirds (approx. 4000 species) represent nearly 50% of all avian species and they offer great opportunities for studies in social behavior (Davies, 1992; Alcock, 2009; Székely et al., 2010). Songbirds have diverse mating systems and parental care that attracted seminal studies (Lack, 1968; Bennett and Owens, 2002). Although many songbirds are socially monogamous and both the male and the female look after the eggs and young, there are substantial variations from this general pattern. First, only one parent, usually the female may look after the young whereas the males are polygamous and mate with numerous females in a single breeding season, for instance in birds of paradise (Lack, 1968). Second, cooperative breeding, i.e., systems where not only the biological parents but additional individuals (i.e., helpers from nearby territories or from previous batches of reproduction of the focal parent) help rearing the offspring (Feeney et al., 2013). Third, parents may forgo care and deposit their eggs into the nests of other species so that the eggs and the young are cared for by the host parents themselves (brood parasites, e.g., widowbird, Moskat et al., 2010; Feeney et al., 2013). Finally, mating systems and parental care may vary within a single breeding population so that several patterns coexist simultaneously. For instance, in European dunnocks (*Prunella modularis*) males and females may pair monogamously, a single male may have several females (polygyny), a single female may have several males (polyandry) and multiple pair bonds by both the male and female (polygyandry, Davies, 1992).

Whilst behavioral ecologists have documented extensive variations in mating system and parental care within and between bird species, the neural causes of these variations are largely unknown (Mcgraw et al., 2010). Amongst the numerous neuroendocrine and neurotransmitter substances involved in the control of the reproductive behavior of birds, two peptidergic systems have been shown to have important modulatory roles: vasotocinergic and VIP-ergic. Extensively studied in quails, (Balthazart et al., 1997; Panzica et al., 1997) and domestic chicks (Jurkevich and Grossmann, 2003), the vasotocinergic system presents a strong sexual dimorphism (Jurkevich et al., 1997; Panzica et al., 1997) and its activity is under the control of sexual steroid (Balthazart et al., 1997; Panzica et al., 1997; Aste et al., 2013). In songbirds, vasotocin, together with VIP, has been shown to modulate the courtship behavior (Goodson, 1998a,b). The social organization of songbirds has been linked to the activity of the vasotocinergic neurons in the limbic circuit (Goodson, 2008; Goodson et al., 2012a). In addition, AVT facilitates, whereas VIP inhibits the agonist song (directed toward conspecifics) in songbirds (Goodson, 1998a). Changes in VIP immunoreactivity, as well as in VIP receptor gene expression have been identified in the hypothalamus of pigeons and hens in relation to the reproductive cycle (Cloues et al., 1990; Chaiseha et al., 2004). A recent study in zebra finch underlines the activation of VIP expression during nesting behavior (Kingsbury et al., 2015). VIP is also modulating the nesting behavior in hens and turkeys (Macnamee et al., 1986; Prakobsaeng et al., 2011). It should be noted that VIP has been shown to be the prolactin releasing neurohormone in birds responsible for initiating incubation behavior (Macnamee et al., 1986).

Of the common and well-studied European passerines, here we focus on the blue tit [*Cyanistes coeruleus*, formerly *Parus coeruleus* (Johansson et al., 2013)]. European blue tits are small birds (body mass approx. 11 g), widely used as a model species in studies of ecology, behavior, and evolution. The numerous investigations cover a wide variety of domains such as phenology (Massa et al., 2011; Matthysen et al., 2011), and behavioral studies (Foerster and Kempenaers, 2004; Aplin et al., 2013). Living in flocks during the winter, blue tits pair at the beginning of the breeding season (April or May). The pair remains together throughout the breeding season or even across several breeding seasons, although pair bonds are unstable (Pampus et al., 2005; Valcu and Kempenaers, 2008). Both parents care for the chicks (Dickens and Hartley, 2007). Although socially monogamous, it is a facultative polygynous bird, and extrapair paternity may range from 31 to 65% (Kempenaers et al., 1997; Valcu and Kempenaers, 2008).

Despite the numerous studies on behavior, ecology and life history of tits, only limited data are available on their neuroanatomy. Some morphometric studies have compared the relative volume of the blue tit hippocampus with that of food storing birds (Healy and Krebs, 1996). Apart from our previous work, restricted to the AVT-like and VIP-like immunoreactivity in some limbic nuclei of the social brain network in the blue tit and the penduline tit (Montagnese et al., 2014), we found only one study describing the distribution of NPY and Substance P in hippocampal areas comparing different species of wild passerine birds (Gould et al., 2001). Thus, comparative studies on neuropeptide distribution in wild birds are generally scarce. The current report is intended to fill the existing gap in the neuroanatomy of songbirds, probably representing the only detailed mapping study available in Paridae. The aim of the present article is to extend our knowledge by the mapping of vasotocin and VIP systems in the brain of the blue tit, enabling further studies of behavior associated neuroanatomy and neuroendocrinology.

## Materials and methods

### Animals

Five male and two female blue tits (*Cyanistes coeruleus*), all adults, were caught at their nests, in the Dnestr Delta National Park, Ukraine, between 21 May 2009 and 26 May 2009, by using a mist net and by playing species specific songs as bait. The research was approved by the Ministry of Environmental Protection of the Ukraine and the National Park of Lower Dnestr Region. The work was carried out in accordance with the Directive 2010/63/EU of the European Parliament and of the Council on the protection of animals used for scientific purpose.

After ketamine-xylazine anesthesia, the brains were dissected out and immediately fixed by immersion in a solution of 4% paraformaldehyde in 0.1 M phosphate buffer. Samples were stored at 4°C until further processing. Fixed brains were transferred to a 20% sucrose solution before being sectioned at a 60 μm thickness on a freezing microtome (Frigomobil, Zeiss). Three series of alternate sections were taken. One series was immediately mounted and stained with cresyl violet for identification of the structures. Two other series were processed for immunocytochemistry.

### Immunohistochemistry

Two antisera were used for this study, an anti-AVT (kind gift from Prof. David Gray University of Witwatersrand, Johannesburg, RSA, Gray and Simon, 1983) and an anti-VIP (gift from Dr. Tamás Görcs, Gulyas et al., 1990). Both were raised in rabbit and diluted in PBS-Tween 20 (anti-AVT: 1:60000, anti VIP: 1:10000).

The AVT antiserum proved to be specific for the measurement of AVP in dogs and AVT in ducks using radioimmunoassay, while the method and standards of antibody generation are also given by the cited paper (Gray and Simon, 1983). In our hands, in preliminary tests on zebra finch brain, the antiserum could be diluted up to 1:150 000, in order to achieve total extinction of the staining. Preabsorption with AVT peptide prevented staining, however, preabsorption with either oxytocin or mesotocin did not abolish staining.

The VIP antiserum was generated from a synthetic VIP conjugated to bovine thyroglobulin with glutaraldehyde as a cross-linking reagent. The antibody was tested for cross-reactivity with related peptides and VIP staining was eliminated by preabsorption with human, porcine and rat VIP (Gulyas et al., 1990). In a subsequent radioimmunoassay study, the specificity of VIP antiserum was tested in a number of species, including the chicken (Nemeth et al., 2002).

Sections were washed in PBS. Endogenous peroxidase activity was quenched by 0.1% H_2_O_2_ in PBS for 15 min. Following several washes in PBS containing 0.1% Tween 20 (Sigma-Aldrich, Steinheim, Germany), sections were incubated for 2 h in a solution of 1% normal goat serum in PBS-Tween 20, and then transferred overnight to the rabbit primary antiserum. Then, sections were extensively washed in PBS-Tween 20, incubated for 2 h in a biotinylated goat anti-rabbit IgG (Vector, Burlingame, CA) at 1/100 in PBS-Tween-20, rinsed and incubated with avidin-biotin complex (Vector, Burlingame CA) diluted in PBS for 2 h. Sections were rinsed first in PBS, then in Tris buffer (pH 8), before being incubated in a solution containing 0.015 % of diaminobenzidine tetrahydrochloride (DAB, Sigma-Aldrich, Steinheim, Germany) and 0.25% ammonium nickel sulfate hexahydrate (Fluka Chemie, Buchs, Switzerland) in Tris buffer. After 5-min preincubation, the enzymatic reaction was initiated by adding 5 μl H_2_O_2_ (0.1%)/5 ml DAB solution. The reaction was stopped 10 min later by rinsing with Tris buffer, followed by PBS. Sections were then mounted on gelatin-subbed slides and coverslipped with DPX (Sigma-Aldrich, Steinheim, Germany). Control of specificity included omission of the primary antisera, and absorption of the antiserum with the antigen. Non-specific staining was not observed in the tissue.

### Identification of the brain structures

Contour drawings of the Nissl-stained sections served as templates on which the AVT and VIP immunoreactive cells, fibers and terminal fields were recorded. In general, we applied the terminology outlined by the Avian Nomenclature Forum (Reiner et al., 2004). In addition, for identification of specific brain regions, we also used the canary atlas (Stokes et al., 1974) and the chicken atlases (Kuenzel and Masson, 1988; Puelles et al., 2007). For the septal areas, we identified the different subdivisions as defined by Goodson et al. (2004). Certain hypothalamic subgroups of the AVT+ neurons were identified according to the terminology of Berk (Berk et al., 1982). To compose the table, the relative abundance of the immunoreactive elements was visually estimated and classified as occasional (not systematically present or sporadic), few, moderate and numerous; whereas the variability of abundance between birds is represented by a “/” between the smallest and highest estimate.

## Results

**Figure 1:**
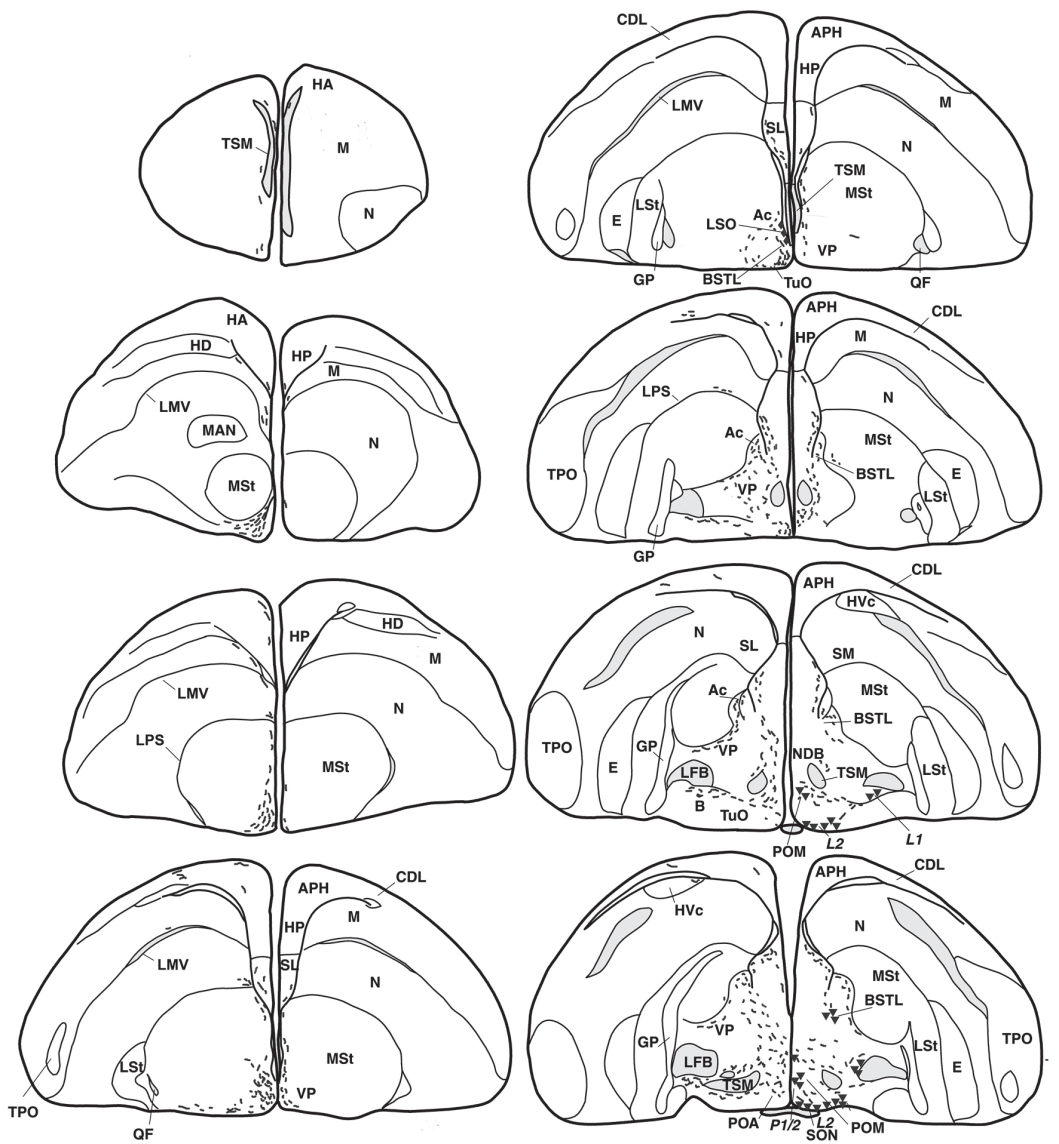
**Representative drawings of the distribution of vasotocine-like immunoreactive (right half of the brain) and vasoactive intestinal peptide-like immunoreactive (left) perikarya (triangles and diamonds, respectively) and fibers (short lines) throughout the brain of the blue tit** (***Cyanistes coeruleus***). [A, arcopallium; A8, dopaminergic cell group; Ac, nucleus accumbens; AL, ansa lenticularis; AM, hypothalamic anterior medial nucleus; Am, medial arcopallium; AP, pretectal area; APH, parahippocampal area; B, magnocellular nucleus basalis; BC, brachium conjunctivum; BSTL, lateral part of the bed nucleus of the stria terminalis; CA, anterior commissure; CDL, dorsolateral corticoid area; CHCS, corticohabenular and corticoseptal tract; CO, optic chiasm; CP, posterior commissure; DBC, decussation of the brachium conjunctivum; *DD1-DD2*, dorsal diencephalic vasotocinergic cells groups (Berk et al., 1982); DHA, dorsal hypothalamic area; DLA, anterior dorsolateral thalamic nucleus; DLP, posterior dorsolateral thalamic nucleus; DMA, anterior dorsomedial thalamic nucleus; DMN, medial dorsal hypothalamic nucleus; DMP, posterior dorsomedial thalamic nucleus; DSV, ventral supraoptic decussation; E, entopallium; EW, nucleus of Edinger–Westphal; FLM, medial longitudinal fascicle; FRL, lateral mesencephalic reticular formation; FRM, medial mesencephalic reticular formation; GCt, central gray; GLv, ventral part of the lateral geniculate nucleus; GP, globus pallidus; HA, apical part of the hyperpallium; HD, densocellular part of the hyperpallium; HL, lateral habenular nucleus; HM, medial habenular nucleus; HP, hippocampal formation; HVC, higher vocal center; ICo, intercollicular nucleus; IMc, magnocellular part of the isthmic nucleus; IP, interpeduncular nucleus; IPc, parvocellular part of the isthmic nucleus; *L1-L4*, hypothalamic vasotocinergic cells groups (Berk et al., 1982); LA, lateral anterior thalamic nucleus; LMV, lamina mesopallialis ventralis/lamina mesopallialis; LC, nucleus linearis caudalis; LFB, lateral forebrain bundle; LM, mesencephalic nucleus lentiformis; LoC, locus coeruleus; LPS, lamina pallio-subpallialis; LSO, lateral septal organ; LSt, lateral striatum; LHy, lateral hypothalamic area; M, mesopallium; MAN, magnocellular nucleus of the nidopallium; ME, median eminence; MLd, dorsal part of the lateral mesencephalic nucleus; MnV, motor nucleus of the trigeminal nerve; MSt, medial striatum; N, nidopallium; nCPa, nucleus of the pallial commissure; NDB, nucleus of the diagonal band of Broca; NIII, oculomotor nerve; nIV, nucleus of the trochlear nerve; OM, occipitomesencephalic tract; OMd, dorsal part of the oculomotor nucleus; OMv, ventral part of the oculomotor nucleus; Ov, nucleus ovoidalis; *P-P1-P3*, hypothalamic periventricular vasotocinergic cells groups (Berk et al., 1982); PHN, periventricular hypothalamic nucleus; PMI, internal paramedian thalamic nucleus; POA, preoptic area; POM, magnocellular preoptic nucleus; PPT, pedunculopontine tegmental nucleus; PrV, principal trigeminal nucleus; PT, pretectal nucleus; PTD, diffuse pretectal nucleus; PTM, medial pretectal nucleus; PVN, paraventricular hypothalamic nucleus; PVO, periventricular organ; QF, quintofrontal tract; R, raphe nuclei; RA, robust nucleus of the arcopallium; ROT, nucleus rotundus; RP, pontine reticular formation; Ru, red nucleus; SCd, dorsal subcoeruleus nucleus; SCN, suprachiasmatic nucleus; SCv, ventral subcoeruleus nucleus; SGP, stratum griseum periventriculare; SL, lateral septum; SM, medial septum; SN, substantia nigra; SNc, substantia nigra pars compacta; SON, supraoptic nucleus; SP, subpretectal nucleus; SPL, lateral spiriform nucleus; StPaAc, striatopallidal area of the nucleus accumbens of Puelles; TeO, optic tectum; TnA, nucleus taenia of the amygdala; TPO, temporo-parieto-occipital area; TSM, septopallial mesencephalic tract; Tu, tuberal/arcuate nucleus; TuO, olfactory tubercle; VeM, medial vestibular nucleus; VLT, ventrolateral thalamic nucleus; VMN, ventromedial hypothalamic nucleus; VP, ventral pallidum; VTA, ventral tegmental area; X, area X; ZI, zona incerta].

**Figure 2:**
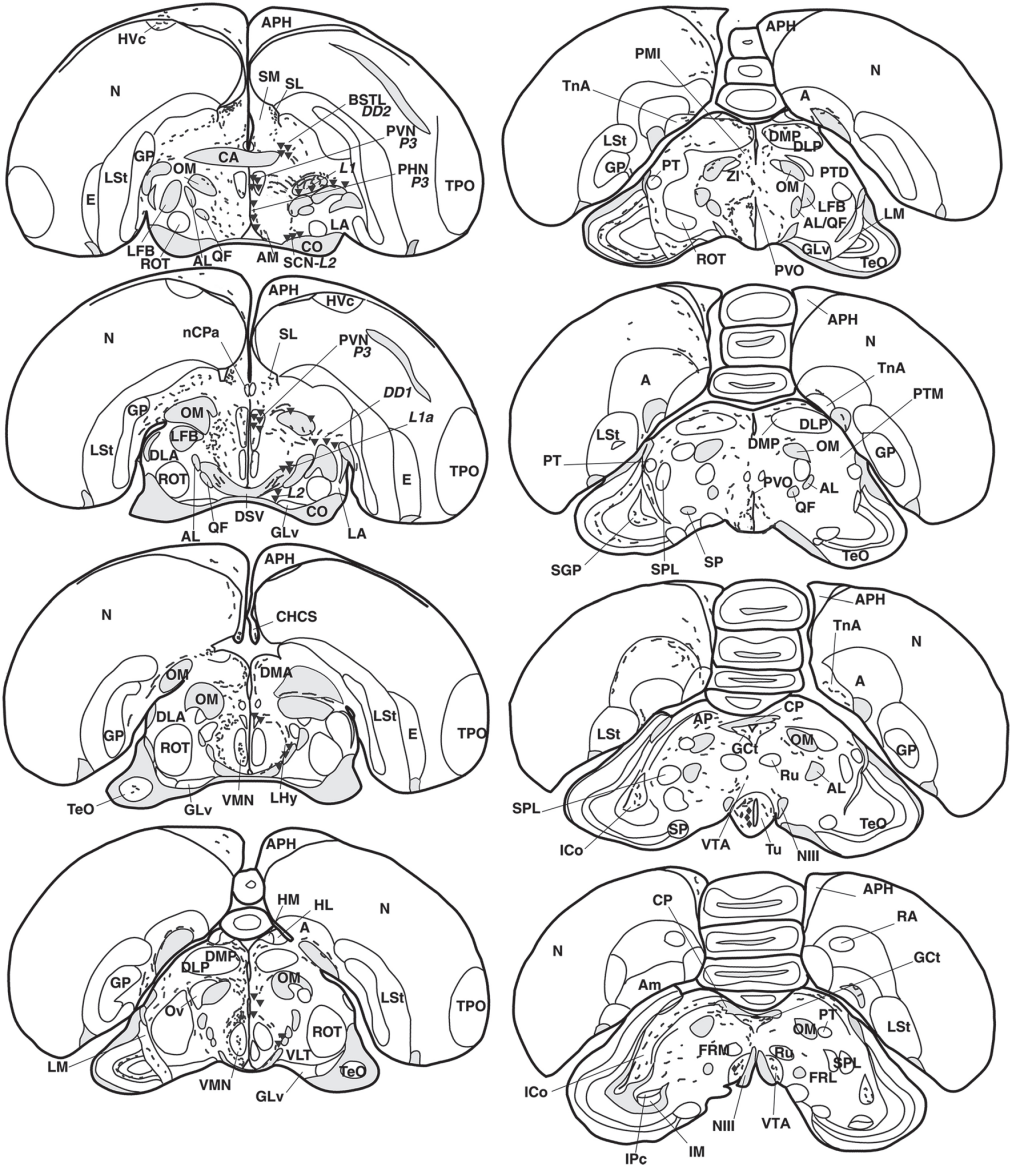
**Refer Figure 1 caption**.

**Figure 3:**
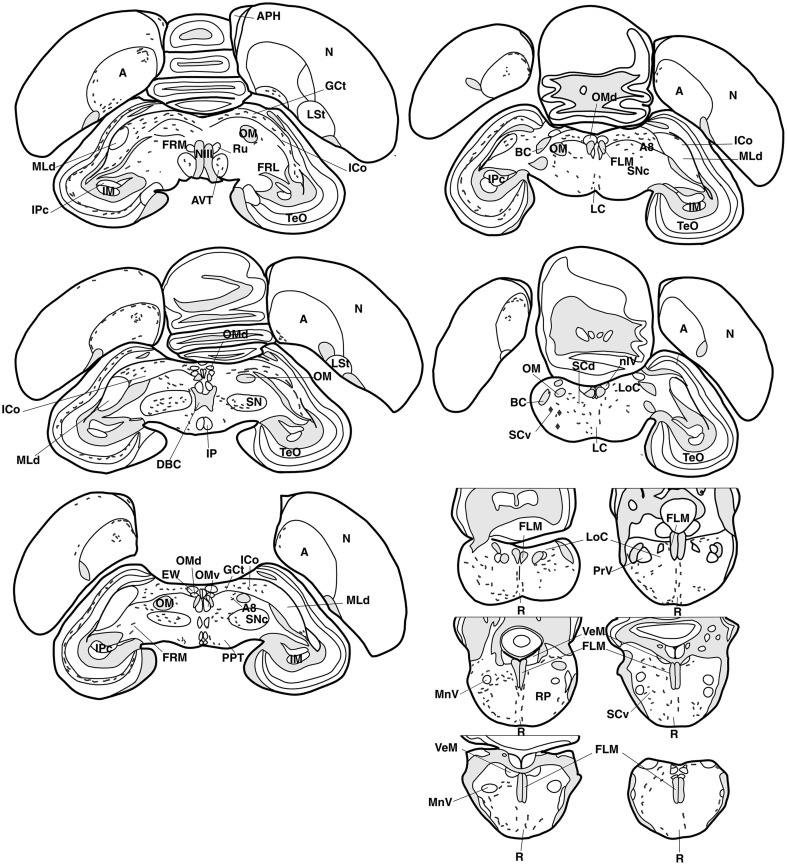
**Refer Figure 1 caption**.

Anatomical distribution of the neural elements immunoreactive to Vasotocine or VIP is shown on sequential sets of diagrams (Figures 1–3). The diagrams are based on contour drawings of original histological specimens, rather than atlas templates. On each drawing VIP labeled structures are mapped on the left side, and AVT labeled structures on the right side. Each set of diagrams represents an individual case of bird, while composite results are shown in Table 1. We show only semi-quantitative data of distribution in both sexes in a tabulated form, since the number of cases was considered too low for an intersexual comparison in quantitative terms.

**Table 1:** **Distribution of VIP- and AVT-immunoreactive neurons, fibers and terminal fields in the brain of male and female blue tits**.

	**VIP immunoreactivity**				**AVT immunoreactivity**			
	**PC_♂_**		**PC_♀_**		**PC_♂_**		**PC_♀_**	
	**Cells**	**Fibers and terminals**	**Cells**	**Fibers and terminals**	**Cells**	**Fibers and terminals**	**Cells**	**Fibers and terminals**
**TELENCEPHALON**								
**Pallium**								
Hyperpallium apicale		+		+				
Densocellular part of the hyperpallium/mesopallium dorsale (Jarvis)				+				
Mesopallium/mesopallium ventrale (Jarvis)								
*medial periventricular*		+/++		+/++				+
*medial and central part*		−/+		−/+				
*caudal (HVc)*		+/++		−/++				
Dorsolateral corticoid area		+/++		+				
Nidopallium								
*frontal periventricular*		+		+				
*medial periventricular ventral*		+/++		+/++				
*intermediate*								
*central part*				+				
*periventricular ventral*		+/++		+/++				
*Field L (part of intercalated nidopallium)*		+/++		+/++				
*caudal medial*		+/++		+				
*caudal lateral*		+		+				
Hippocampal formation		+		+				+
Parahippocampal area		+		+				
Arcopallium		++/+++		+/++		++		+
*Anterior*		++		++				
*Medial*		++/+++		+/++		++/+++		+
*Robust Nucleus shell*		+++						
*Posterior*						+		
*Ventral*		++						
*Nucleus taeniae of the amygdala*		++/+++		++		+/++		+
**Subpallium**								
***Striatal subpallium***								
Medial striatum		+		+				+
Nucleus accumbens								
*Striatopallidal (Puelles)*		+++		+++				
*rostral pole*		++		++		+/++		−/++
*shell*		++		++		−/++		
*core*		+/++		+/++		+		
***Pallidal subpallium***								
Globus pallidum				+				
Ventral pallidum		+++		++/+++		++/+++		+/++
Bed nucleus of the stria terminalis								
*lateral part*		+/++		++/+++		++/+++		+/++
*ventromedial part*		+/++		++/+++	++/+++	++/+++	++/+++^1^	++/+++
*dorsolateral part*		+/+++		+++	+++/++++	+++/++++	++	++
*magnocellular part*		+/++		+		+		+
Basal nucleus (Meynert)		++/+++		++/+++		+		
***Striatopallidal subpallium***								
Olfactory tubercle								
*medial TuO*		++/+++		++/+++				+/++
*lateral TuO*		+/++		++				
***Septal subpallium***								
Lateral septum								
*rostral "pole"*		++/+++		++/+++		+/+++		+
*rostral SLc.v*		++/+++		++/+++		++/+++		+
*rostral SLc.vl*		++/+++		+++		+/+++		+
*SLr.dl*		+/++				+/+++		+
*SLr.m*		+/++				−/+++		
*SLc.d*		+/++		++		++/+++		
*SLc.v*		++/++++		++/++++		++/+++		+/++
*ccs*		−/+++		+++		++/+++		−/++
Medial septum								
*medial septal nucleus*		+						
*intermediate band of the medial septum*		+/++		+/++				
Nucleus of the diagonal band		++/+++		+/++		++/+++		+
Septal commissural nucleus		−/+++		+/++		+/++		+
Corticohabenular and corticoseptal tract		++/+++						+
Nucleus of the pallial commissure		+/+++		+/++		−/++		+
Lateral septal organ	+++	+++	+++	+++				
Preoptic area		++/+++		++/+++	+/++++	+++/++++	++/++++^1^	+++/++++
Medial preoptic nucleus		++/+++		++/+++	++++	+++/++++	+++/++++^1^	+++/++++
Periventricular preoptic nucleus					++/+++		++/+++	
**Tracts**								
Lamina frontalis superior/lamina mesopallialis dorsalis		+		+				
Lamina pallio-subpallialis		+		+				
Septomesencephalic tract		+						
Ventral amygdalofugal tract		++		++				
**DIENCEPHALON**								
**Thalamus**								
Thalamic anterior dorsomedial nucleus		+++		++/+++		+++		+++
Thalamic posterior dorsolateral nucleus		−/++		−/++				
Thalamic posterior dorsomedial nucleus		+++		++/+++		+++		++/+++
Thalamic ventroanterior dorsointermediate nucleus		−/++						
Thalamic nucleus paramedianus internus		++		++/+++		++		++/+++
Intermediate periventricular nucleus (Puelles)		++		−/++		+/+++		−/++
Thalamic ventrolateral nucleus		−/++		−/++				
Ventral periventricular nucleus (Puelles)		++		++		++		−/++
Nucleus reticularis superior dorsal part		−/++						
Intergeniculate leaflet		−/++		−/++				
Nucleus of the septo-mesencephalic tract		++		++				
Lateral habenula		++/+++		++/+++		+		+
**Prethalamus**								
Zona incerta		+/++		++				
A13 area		++/+++		++		+/++		+
**Hypothalamus**								
Ventral supraoptic nucleus					+++		+++	
External supraoptic nucleus					+/+++		++/+++	
Suprachiasmatic nucleus					+++		+++	
Hypothalamic anterior nucleus		++		++				
Lateral hypothalamic area		++/+++		+/+++	+++	+++	+++	+++
Hypothalamic periventricular nucleus				−/++	++/+++	++/+++	++/+++	++/+++
Paraventricular nucleus		++/+++		++/+++	++++	+++/++++	++++	++++
Hypothalamic ventromedial nucleus (core)		++/+++		++/++++				
Hypothalamic dorsomedial nucleus		++/+++		++/+++				
Hypothalamic dorsal area		++		++/+++		+/++		+
Hypothalamic inferior nucleus	+++	++/+++	++++	++/++++				
Infundibular nucleus	++++	+++/++++	++++	+++/++++				
*Arcuate (Tuberal) nucleus*	++++	+++/++++	++++	+++/++++				
**BRAINSTEM**								
**Pretectum/mesencephalon**								
Mesencephalic nucleus lentiformis		++		++				
Diffuse pretectal nucleus		++		++		+		+
Pretectal area		++		++		+		+
Perirubral region	+	+						
Midbrain central gray		++/+++		++/+++		+/+++		++
Intercollicular nucleus		++/+++		++/+++		++/+++		+/++
Mesencephalic lateral reticular formation		+/++		+/++	+	+		
Mesencephalic medial reticular formation		+/++		++		+/++		
Ventral tegmental area	+	++/+++		+++		++/+++		+++
Substantia nigra pars reticulata	+	+/++	+	+/++		+/++		+
Substantia nigra parscompacta		++		++		+/++		+/++
A8		+/++		++		+/++		+/++
**Optic tectum**		++/+++		++/+++				
Stratum griseum centrale		++		++		−/++		
**Rhombencephalon**								
Pedunculopontine tegmental nucleus		+/++		++		+		+
Interpeduncular nucleus		+		+		−/+		+
Locus coeruleus		+/++		++		+/++		++
Dorsal nucleus subcoeruleus		++		++		+		+
Ventral nucleus subcoeruleus	−/+	++		++		+		+
Nucleus linearis caudalis/raphe		++		++		++		++
Pontine reticular formation	+	++/+++	+	++/+++		+/++		+/++
**MEDULLA OBLONGATA**								++

*+: occasional; ++: few; +++: moderate; ++++: numerous; 1. weak immunoreactivity*.

### Vasotocine-like immunoreactivity distribution

#### Distribution of neuronal cell bodies

**Figure 4:**
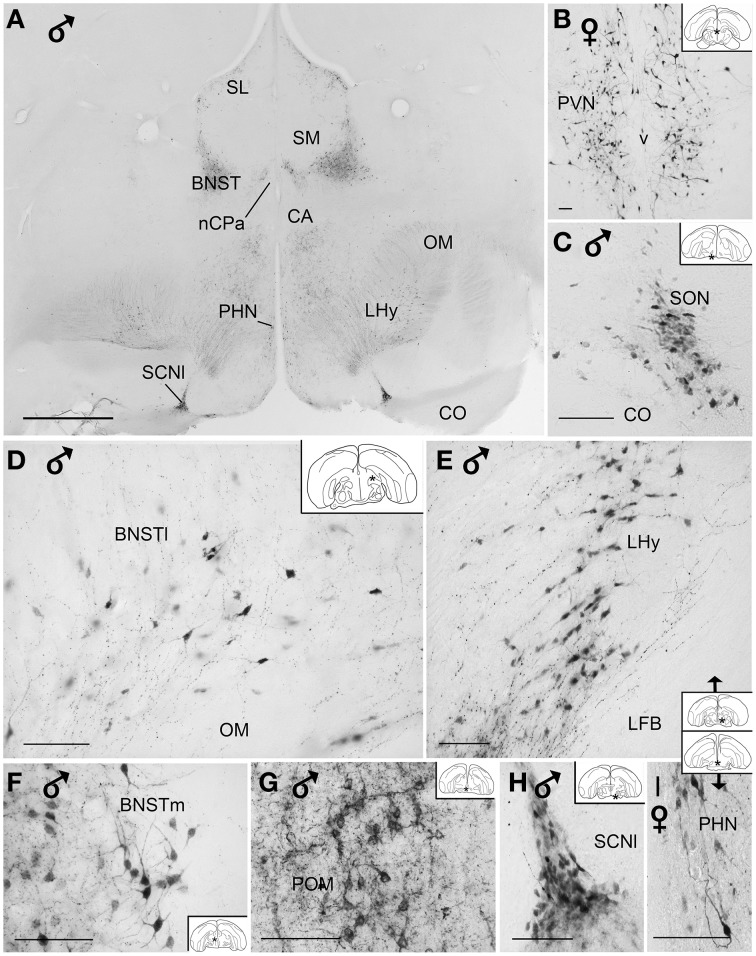
**(A)** Low power photomicrograph showing the distribution of vasotocin-like immunoreactive neurons and fibers in the diencephalon of blue tits. **(B–I)** High power photomicrographs of vasotocin-like immunoreactive perikarya of the brain blue tits. **(B)** hypothalamic paraventricular nucleus, **(C)** supraoptic nucleus, **(D)** bed nucleus of the stria terminalis, pars lateralis, **(E)** lateral hypothalamic area, **(F)** bed nucleus of the stria terminalis, pars medialis, **(G)** medial preoptic nucleus, **(H)** lateral suprachiasmatic nucleus, **(I)** hypothalamic periventricular nucleus. Inset: stars indicate the location of the photographed field on the topogram.Calibration bar 100 μm.

The majority of the AVT-immunoreactive (AVT+) neuronal perikarya were consistently distributed in the hypothalamic ventral supraoptic (Groups *L1, L2*, Figures 1, 4C), suprachiasmatic (Group *L2*, Figures 2, 4A,H), periventricular (Groups *P1-P3*, Figures 1, 2, 4A,I) and paraventricular (Group *P3*, Figures 2, 4B) nuclei (see also Table 1). Many perikarya were dispersed amongst the fibers of the lateral forebrain bundle and lateral hypothalamic area (Group *L1*, Figures 2, 4E, Table 1). Most of these neurons were multipolar, more rarely bipolar.

AVT+ neurons were also located in two sexually dimorphic nuclei: the medial preoptic and periventricular nuclei (group P1/2, Figures 1, 4G, Table 1) and the medial bed nucleus of the stria terminalis (Group *DD2*, Figures 2, 4F). Some AVT+ neurons present in the thalamus, on the lateral side of the lateral forebrain bundle (Group *DD1*), most probably correspond to the bed nucleus of the stria terminalis pars lateralis (Figures 2, 4D). In some birds, the preoptic neurons were continuous with those of the paraventricular nucleus.

#### Distribution of the fibers and terminals

##### Hypothalamic fibers

**Figure 5:**
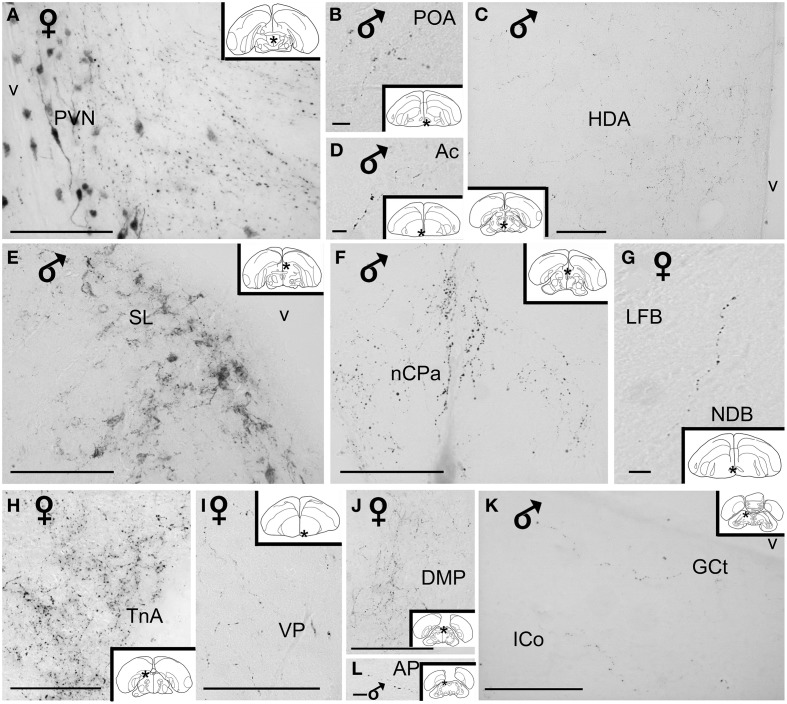
**High power photomicrographs of vasotocin-like immunoreactive fibers and terminal fields in the brain of the blue tit. (A)** hypothalamic paraventricular nucleus, **(B)** preoptic area, **(C)** hypothalamic dorsal area, **(D)** nucleus accumbens, **(E)** lateral septum, **(F)** nucleus of the pallial commissure, **(G)** nucleus of the diagonal band of Broca, **(H)** nucleus taeniae of the amygdala, **(I)** ventral pallidum, **(J)** thalamic dorsomedial posterior nucleus **(K)** mesencephalic central gray and intercollicular nucleus, **(L)** pretectal area. Inset: stars indicate the location of the photographed field on the topogram. Calibration bar 100 μm.

Hypothalamic AVT+ neurons gave rise to axons coursing essentially through the lateral hypothalamus into the hypothalamic-hypophyseal tract toward the median eminence (Figures 1, 2, 4A), including those originating from the paraventricular nucleus (Figure 5A). Other fibers followed a periventricular course. A few fibers were also crossing through the supraoptic decussation toward the contralateral hypothalamus. Fibers were occasionally observed in the hypothalamic dorsal area (Figure 5C) and the dopaminergic A13 region. (See also Table 1).

##### Extrahypothalamic fibers

###### Subpallium

The main extrahypothalamic sites of labeling comprised the bed nucleus of the stria terminalis, lateral septal areas and the preoptic region (Table 1, Figures 1, 2, 4D,F,G, 5B,E). In the lateral septum, the AVT+ fibers appear to surround non-immunoreactive neuronal perikarya (Figure 5E). In addition to these areas, some fibers were observed in the nucleus of the diagonal band of Broca (Table 1, Figures 1, 5G), in and around the nucleus of the pallial commissure (Figure 5F) and in the septal commissural nucleus as well as in the periventricular preoptic nucleus (Figure 1).

Beside these areas, AVT+ fibers were relatively frequent in the ventral pallidum (Table 1, Figures 1, 5I). Although rare, they were also encountered in all parts of the nucleus accumbens (Table 1, Figures 1, 5D), medial olfactory bulb, and the basal nucleus of Meynert (Table 1).

###### Pallium

Rare AVT+ fibers and terminals were present in the arcopallial centers, mainly the medial arcopallium and the nucleus taeniae (Table 1, Figures 2, 5H). They tended to be more frequent in the male than in the female (Table 1). Occasionally AVT+ fibers were observed in the hippocampal formation (Figures 1–3) and the periventricular medial mesopallium (Table 1).

###### Thalamus

Many AVT+ fibers terminated in the medial part of the thalamic anterior and posterior dorsomedial nuclei (Figure 5J), the thalamic nucleus paramedianus internus and the intermediate periventricular nuclei of Puelles et al. (2007) (Table 1, Figure 2). They were less numerous in the ventral periventricular nucleus of Puelles et al. (2007) as well as in the stratum cellulare internum (coextensive with A13) (Table 1, Figure 2). Single fibers entered the lateral habenula (Table 1, Figure 2).

###### Brainstem

AVT+ fibers were mostly present in the midbrain central gray (Figure 5K), intercollicular nucleus (Figure 5K), ventral tegmental areas, and the nucleus linearis caudalis (Table 1, Figure 3). Less frequent AVT+fibers were observed in the mesencephalic and pontine reticular formations, both compact and reticular parts of the substantia nigra, A8 and locus coeruleus (Figures 2, 3). Occasional AVT+ fibers were observed in the pretectal areas (Figure 5L), pedunculopontine tegmental nucleus, dorsal and ventral subcoeruleus nuclei (Table 1, Figures 2, 3). AVT+ fibers also descended in the medulla oblongata in the limited number of cases where this region was preserved (Table 1).

### Vasoactive intestinal peptide-like immunoreactivity distribution

#### Distribution of neuronal cell bodies

**Figure 6:**
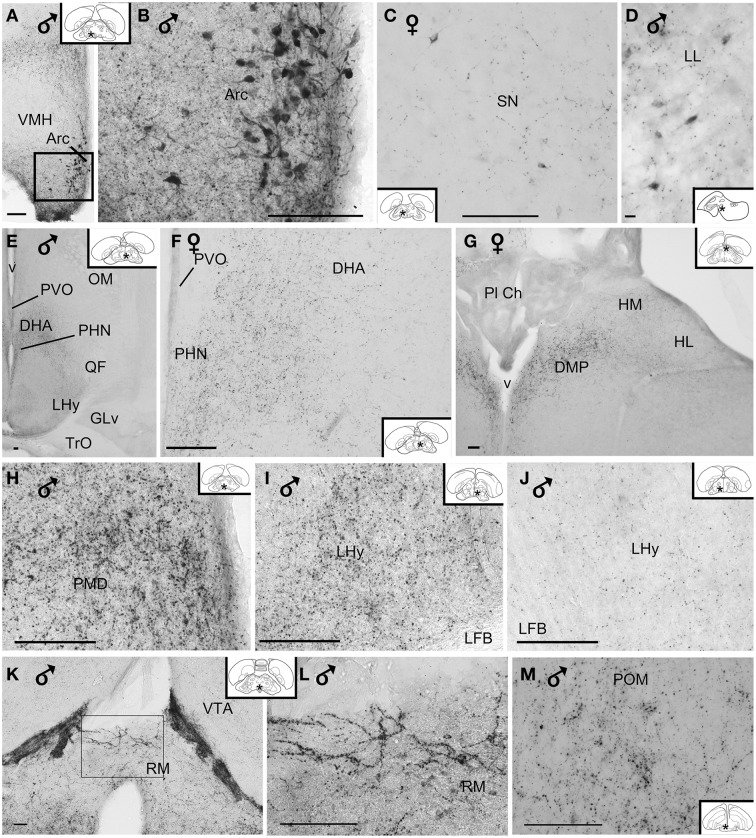
**(A–D)** Photomicrographs of vasoactive intestinal peptide-like immunoreactive perikarya in the brain of blue tit: **(A,B)** arcuate nucleus, **(C)** substantia nigra, **(D)** lateral lemniscus. **(E)** Overview image of the hypothalamus. Fibers and terminal field **(F)** in the dorsal hypothalamic area, **(G)** posterior dorsomedial thalamus and habenular nuclei, **(H)** dorsal premammillary nucleus, **(I,J)** lateral hypothalamic area showing variability of immunoreactivity between individuals, **(K,L)** retromammillary area, **(M)** medial preoptic nucleus. **(B,L)** Are enlargements of the areas framed in **(A,K)**, respectively. Inset: stars indicate the location of the photographed field on the topogram. (Abbreviations in addition to those listed in the legend to Figure 1: LL, lateral lemniscus; Pl Ch, choroidal plexus; RM, retromammillary area). Calibration bar 100 μm **(A–C,E–M)**, 10 μm **(D)**.

Numerous neuronal cell bodies were immunoreactive for VIP in the lateral septal organ (Figure 1), the hypothalamic inferior and arcuate nuclei of all birds (Figures 2, 6A,B) see also Table 1.

A few VIP+ neurons were observed in the ventral tegmental area, substantia nigra (Figure 6C), pontine reticular formation, perirubral region, amongst the lateral lemniscus bundles (Figure 6D) and, in one bird, the ventral subcoeruleus nucleus (Figure 1).

#### Distribution of the fibers and terminals

Although the density of the VIP-immunoreactive (VIP+) fibers may vary considerably between individuals (as shown between two male tits in the lateral hypothalamic area in Figures 6I,J), within any given subject the relative density between areas remains consistent.

##### Hypothalamic fibers

VIP+ fibers were distributed extensively in the hypothalamus (Figure 6E). As shown in Table 1 and Figure 2, the highest density of VIP+ fibers and terminal fields were found in the lateral hypothalamic area (Figures 6F,I,J), hypothalamic ventromedial nucleus core, paraventricular nucleus, infundibular/tuberal nuclei including the arcuate nucleus (Figures 6A,B) and hypothalamic inferior nucleus, as well as the mammillary, premammillary and retromammillary area (Figures 6H,K,L). The density of VIP+ fibers was modest in the dorsal hypothalamic area (Figure 6F), hypothalamic anterior nucleus and hypothalamic periventricular nucleus (Figure 6F).

##### Extrahypothalamic fibers

###### Subpallium

**Figure 7:**
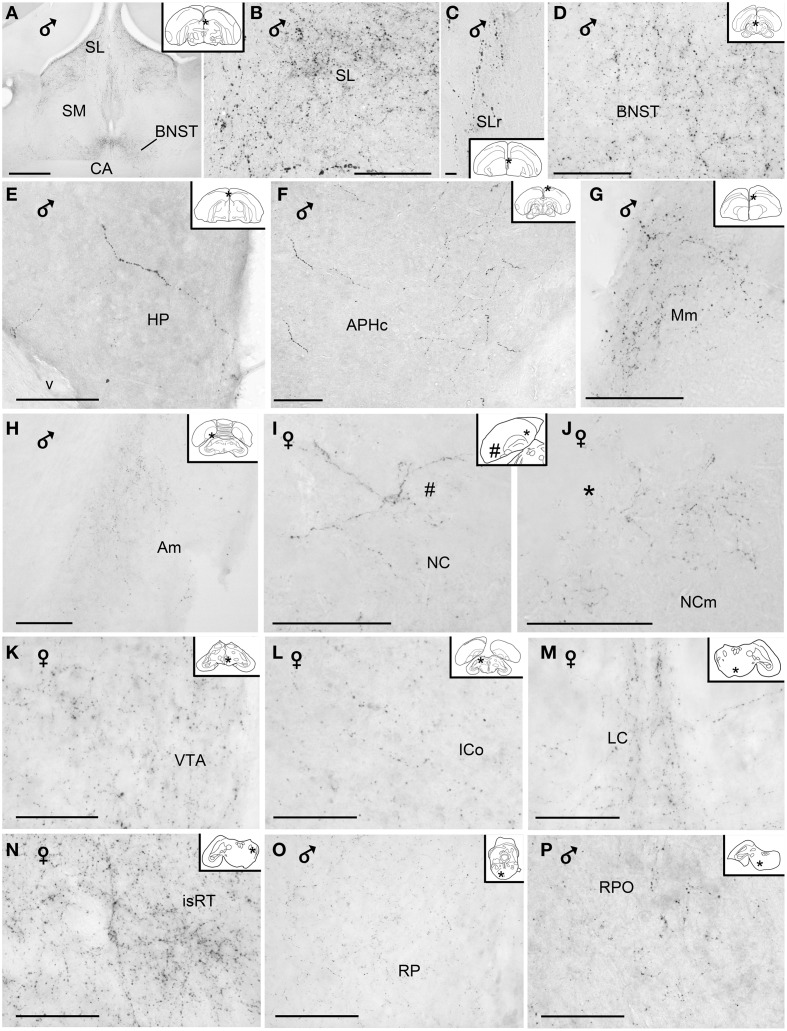
**Photomicrographs of extrahypothalamic vasoactive intestinal peptide-like immunoreactive fibers and terminal fields. (A)** Overview of the septal areas at intermediate level, **(B)** lateral dorsal septum, **(C)** rostral lateral septum, **(D)** bed nucleus of the stria terminalis, **(E)** hippocampal formation, **(F)** caudal parahippocampal area, **(G)** medial mesopallium, **(H)** medial arcopallium, **(I)** lateroventral caudal nidopallium, **(J)** mediodorsal caudal nidopallium, **(K)** ventral tegmental area, **(L)** intercollicular nucleus, **(M)** nucleus linearis caudalis, **(N)** isthmic reticular formation, **(O)** pontine reticular formation, **(P)** oral pontine reticular nucleus. (Abbreviations in addition to those listed in the legend to Figure 1: cAPH, caudal parahippocampal area; Mm, medial mesopallium; isRT, isthmic reticular formation; NC, caudal nidopallium; NCm, medial caudal nidopallium; RPO, oral pontine reticular nucleus; SLr, rostral lateral septum; v, ventricle). Inset: stars indicate the location of the photographed field on the topogram. On the joint topogram of **(I,J)**, the location of the photograph of **(I)** is marked by a #, and that of **(J)** by a ^*^. These symbols also appear on the respective images. Calibration bar 100 μm **(A,B,D–P)**, 10 μm **(C)**.

The majority of the VIP+ fibers terminated in the lateral septal region, while in the medial septal areas they were mainly confined to the intermediate band of the medial septum, seemingly avoiding the medial septum itself (Table 1, Figures 1, 2, 7A–C). In the lateral septal areas, the greatest density of VIP+ fibers and terminals were found in the ventral and ventrolateral parts of the caudal lateral septum (Table 1). In some cases, VIP terminals completely surround and perhaps contact non-immunoreactive perikarya in the lateral septal nucleus. VIP+ fibers also terminated in the nucleus of the diagonal band of Broca (Table 1, Figure 1). Some fibers were present in the commissural septal nucleus, with a great variation between birds (Table 1).

Numerous VIP+ fibers were coursing through and terminating in all subdivisions of bed nucleus of the stria terminalis, including the lateral part of the rostral bed nucleus of the stria terminalis along the ventral part of the lateral ventricle (Table 1, Figures 1, 2, 7A,D). VIP+ fibers were also observed in the preoptic nuclei and area, in particular in the medial preoptic nucleus (Table 1, Figures 1, 6M). Many VIP+ fibers and dense terminal fields were present throughout the nucleus accumbens (Table 1, Figure 1). These fibers were always numerous in the rostral striatopallidal part of the nucleus accumbens (Table 1, Figure 1). In the shell and the core of the nucleus accumbens, the density of VIP+ fibers was more variable (Table 1). These groups of fibers followed a crescent-shaped pathway into the ventral pallidum, where they terminated in a dense field (Table 1, Figure 1). Fibers were also present ventrally to the lateral forebrain bundle, in the nucleus basalis of Meynert and the striatopallidal amygdaloid area of Puelles (Table 1, Figure 1). The medial striatum, including area X, the lateral striatum and the globus pallidus were virtually devoid of VIP staining.

###### Pallial fibers

VIP+ fibers frequently reached the medial arcopallium and the nucleus taenia via the occipitomesencephalic tract (Table 1, Figures 2, 7H). In males, a few fibers run along the medial surface of the arcopallial robust nucleus like a shell. Occasionally fibers were seen in the posterior arcopallium.

Some VIP+ fibers coursed along the septomesencephalic tract and along the lateral wall of the lateral ventricle (Figure 1). These fibers enter the periventricular ventromedial nidopallium immediately dorsally from the palliosubpallial lamina (Table 1), and the periventricular dorso-medial mesopallium (Figure 7G). The mesopallial higher vocal centre, received a greater, though modest, number of VIP+ fibers in males (Table 1, Figure 2). Rare VIP+ fibers coursed lateralward in the ventral telencephalon possibly toward the lateral (striatal) part of the olfactory tubercle along the ventral olfactory tract as identified by Puelles et al. (2007). In the rostral telencephalon, most VIP+ fibers branched in the medial part of the olfactory tubercle. A few VIP+ fibers run along the midline, then the lateral wall of the lateral ventricle, once it has appeared, and enter the medial part of the rostral hyperpallium apicale (Table 1, Figure 1). Rare VIP+ fibers coursed through the hyperpallium apicale, and the hippocampal formation (Figures 7E,F, most of them running along the periventricular layer toward the dorsolateral corticoid area (Table 1, Figures 1–3). Some VIP+ fibers were found in the dorsolateral corticoid area mainly in its medial and rostrocaudal parts (Table 1, Figure 1). Few VIP+ fibers reached the frontal nidopallium following a ventral subpial pathway. Some VIP+ fibers in the rostral lateral nidopallium were restricted to the corticoid plate of Puelles et al. (2007). Moving more caudally, rare VIP+ fibers run along the palliosubpallial lamina and enter the central intermediate nidopallium. More VIP+ fibers were seen in the caudal medial nidopallium at the level of the auditory fields (Table 1, Figures 1, 7I,J).

###### Thalamus

The medial dorsal thalamus (thalamic anterior dorsomedial and thalamic posterior dorsomedial nuclei), a region identified as A13 by Puelles (Puelles et al., 2007), and the periventricular zone were crossed by a moderate number of VIP+ fibers (Table 1, Figures 2, 6G). Some of them coursed along the dorsal surface of the thalamic dorsomedial nuclei lateralward, terminating into the posterior dorsolateral nucleus and the superficial parvicellular nucleus. A few VIP+ fibers were present within the thalamopallial tract, ventral to the dorsomedial thalamus, or the intermediate periventricular and mediocentral nuclei (Puelles et al., 2007) (Table 1). The nucleus paramedianus internus also contained a few VIP+ fibers, with a tendency of being more frequent in females than in males. Other fibers reached the zona incerta (Puelles et al., 2007). Rare VIP+ fibers were observed in the thalamic ventrolateral nucleus, the thalamic posterior and ventroanterior dorsointermediate nuclei, the dorsal part of the nucleus reticularis superior, the ventral lateral geniculate nucleus, the intergeniculate leaflet and the stratum cellulare internum (Table 1).

###### Mesencephalon and brainstem

The main areas containing VIP+ fibers are the midbrain central gray, intercollicular nucleus (Figure 7L), ventral tegmental area (Figure 7K), optic tectum (mainly its stratum griseum centrale), substantia nigra and pontine reticular formation (Figures 7O,P), including isthmic reticular formation (Figure 7N) (see also Table 1 and Figures 2, 3). In all these areas, VIP+ fibers tend to be more frequent in the male than in the female. Fibers were less frequent but consistently observed in several pretectal nuclei (nucleus of the septomesencephalic tract, pretectal nucleus, diffuse pretectal nucleus), perirubral and retrorubral (dopaminergic A8) fields, medial and lateral mesencephalic reticular formation, pedunculopontine tegmental nucleus, locus coeruleus, dorsal and ventral subcoeruleus nuclei, and nucleus linearis caudalis raphes (Table 1, Figures 2, 3, 7M). Scarce VIP+ fibers were observed in the interpeduncular nucleus, dorsal raphe and trapezoid body (Table 1).

## Discussion

We described the distribution of AVT and VIP immunoreactivity throughout the entire brain of blue tits. To our knowledge, this is the first comprehensive study performed in the *Paridae* family, in particular in the *Cyanistes*.

### Notes on methodology

Since the specimens were collected in the wild at various stages of the breeding season this would inevitably generate variability of the anatomical distribution and detectability of these peptides. In our previous work (Montagnese et al., 2014), we focused on the septal and hypothalamic regions in an attempt to find an association between reproductive behavior and neuropeptide distribution in regions described as socially relevant in a wide range of species. However, for any further comparative analysis, it is considered inevitable to provide a thorough and comprehensive mapping of VIP and AVT in those regions where important contingents of peptidergic perikarya or fibers can be detected. Possible intersexual differences were pointed out in our description mainly as a preliminary hint in order to assist further and deeper analysis in the future.

An important question concerning relevance of our data is to what extent can immunoreactivity to a given neuropeptide reflect its cellular metabolism. For the peptides studied here, the correlation between immunodetection and transcription signals is usually good. To our knowledge, no discrepancy between AVT immunoreactive neurons and AVT mRNA has been reported, in fact, similar expression of AVT mRNA and AVT immunoreactive cells was confirmed also in avian species (Jurkevich et al., 1997; Seth et al., 2004; Aste et al., 2013). However, discrepancy between the mRNA of VIP and the detectable VIP immunoreactive perikarya has been reported in the dorsal anterior hypothalamic area of the zebra finch, where mRNA was detectable but VIP immunoreactive perikarya were only visible after colchicine treatment (Goodson et al., 2012b).

### Distribution of vasotocin immunoreactive neurons and fibers and comparison with other avian species

On the basis of topographic and cytological criteria, the AVT+ neurons of the preoptic and diencephalic regions have been categorized as three groups, those belonging to a lateral system (lateral part of the preoptic-hypothalamic region), those belonging to a periventricular system (extending from the preoptic area to the tuberal hypothalamus) and a dorsal diencephalic system (dorsal to the lateral forebrain bundle and the occipitomesencephalic tract) (Berk et al., 1982; Viglietti-Panzica, 1986). The lateral system is composed of the supraoptic, rostral and ventral lateral neurons, the L1 cluster of neurons intermingled with the lateral forebrain bundle, attached to the quintofrontal tract in the extreme lateral preoptic area-hypothalamus; the neuronal cluster L2 ventromedial to L1 on the dorsal border of the optic tract; cluster L3 lying in the lateral preoptic area dorsally to L2 and medially to L1; and two accessory clusters L4 with diffusely arranged cells and L5 with tightly packed neurons respectively located in the lateral and dorsolateral hypothalamus (Berk et al., 1982; Viglietti-Panzica, 1986). The periventricular system is composed of three groups, the rostral medial preoptic P1 lying dorsal to the preoptic recess; and, in a more dorsal position P2 with cells oriented along the axis of the ventricle; followed by P3 at the ventral border of the thalamic anterior dorsomedial nucleus, both of them extending from the vascular organ of the terminal lamina to a postcommissural level, the posterior parts of P2 and P3 correspond to the paraventricular nucleus (Berk et al., 1982; Viglietti-Panzica, 1986). The dorsal diencephalic system includes DD1, a small cluster of cells near the lateral and dorsal borders of the lateral forebrain bundle; and, DD2 a second group on the medial and dorsal surface of the occipitomesencephalic tract in continuity with DD1 and P3 groups (Berk et al., 1982; Viglietti-Panzica, 1986). This group partially overlaps with the bed nucleus of the stria terminalis. In the pigeon, a third group has been identified as DD3 at the ventral border of the magnocellular part of the dorsolateral anterior thalamic nucleus and at the dorsolateral border of the thalamic nucleus rotundus (Berk et al., 1982).

In the blue tit, the vast majority of AVT+ neurons can also be grouped according to the three main systems defined by Berk et al. (1982), those in the preoptic nuclei, in hypothalamic supraoptic, paraventricular and periventricular nuclei and in the bed nucleus of the stria terminalis. This distribution is consistent with earlier observations in other bird species [canary (Kiss et al., 1987), zebra finch (Goossens et al., 1977), domestic fowl (Tennyson et al., 1985; Viglietti-Panzica, 1986), Japanese quail (Goossens et al., 1977; Bons, 1980; Viglietti-Panzica, 1986; Aste et al., 1998), pigeon (Berk et al., 1982), Peking duck (Goossens et al., 1977; Bons, 1980; Viglietti-Panzica, 1986), dark-eyed junco (Panzica et al., 1999), starling (Goossens et al., 1977), and budgerigar (Fabris et al., 2004)]. The lateral system might be less developed in the tits than in the other species, as the L4-L5 groups were not clearly identified. This system is more developed in fowl, duck and pigeon than in Japanese quails (Berk et al., 1982; Viglietti-Panzica, 1986). The periventricular system is equally developed in tits and appears similar to that of the dark-eyed junco (Panzica et al., 1999), starling (Goossens et al., 1977), fowl, duck and pigeon (Viglietti-Panzica, 1986). The extension of the dorsal diencephalic system is known to vary depending on the species, being the largest in fowl (Viglietti-Panzica, 1986). The DD3 group is absent from the tits and so far has only been identified in the pigeon (Berk et al., 1982). In blue tits, the dorsal diencephalic system also appears variable between individuals of the same species.

Two important neuronal groups, the bed nucleus of the stria terminalis and the medial preoptic area, deserve special attention. Both nuclei contain AVT+ neurons and are sexually dimorphic, as already mentioned in our previous work (Montagnese et al., 2014). These nuclear groups have also been observed in male canaries (Kiss et al., 1987; Voorhuis et al., 1988), zebra finches (Voorhuis et al., 1988; Kimura et al., 1999), dark-eyed junco (Panzica et al., 1999), chicken (Jurkevich et al., 1996, 1997), quail (Viglietti-Panzica et al., 1992, 1994; Jurkevich et al., 1996; Panzica et al., 2001) and budgerigar (Fabris et al., 2004). Interestingly, the degree of sexual dimorphism depends on the species. While it is extreme in quail, in that the AVT immunoreactivity is present only in males (Aste et al., 1998), AVT+ neurons also show up in females of other species, such as canaries (Kiss et al., 1987), zebra finches (Voorhuis and De Kloet, 1992), and blue tits (present results). In the present study, weakly stained AVT+ neurons were observed in the sexually dimorphic medial preoptic nucleus of the female blue tit, similarly to previous findings in quail (Panzica et al., 1996; Aste et al., 1997).

With respect to the distribution of AVT+ fibers, the hypothalamic-hypophyseal vasotocinergic system appears to be similar to that of other birds. However this is not the case with other hypothalamic nuclei. Unlike the present findings in tits, vasotocinergic fibers have been identified in the hypothalamic ventromedial nucleus of white-throated sparrows and zebra finches (Leung et al., 2009), dark-eyed juncos (Panzica et al., 1999), Japanese quails (Panzica et al., 2001) and in the lateral mammillary and premammillary nuclei in quails and fowls (Panzica et al., 1988).

Numerous vasotocinergic fibers are present in several extrahypothalamic areas of the tits, including the lateral septum, bed nucleus of the stria terminalis, medial arcopallium, nucleus taeniae of the amygdala, preoptic area, in particular medial preoptic nucleus. These have also been observed in the male canaries (Kiss et al., 1987), quails (Viglietti-Panzica et al., 1992; Aste et al., 1997), zebra finches (Voorhuis and De Kloet, 1992), dark-eyed juncos (Panzica et al., 1999) and budgerigars (Fabris et al., 2004). As described in a previous report (Montagnese et al., 2014), the vasotocinergic fibers of the lateral septal nucleus, bed nucleus of the stria terminalis and medial preoptic nucleus are sexually dimorphic, as in other avian species (Voorhuis et al., 1988; Panzica et al., 1999, 2001). In the telencephalon, at variance with other findings obtained in canaries, zebra finches and dark-eyed juncos (Kiss et al., 1987; Voorhuis and De Kloet, 1992; Panzica et al., 1999), we found fibers in the medial striatum, ventral pallidum, nucleus accumbens, hippocampal formation, but none in the shell and core of nucleus robustus of the arcopallium. Only a few studies have systematically described the distribution of AVT+ fibers in thalamic areas. Such fibers have also been identified in the thalamic posterior dorsomedial nucleus in canaries (Kiss et al., 1987), white-throated sparrows and zebra finches (Leung et al., 2009) and Japanese quails (Panzica et al., 2001). In the nucleus ovoidalis, intermediate periventricular nucleus (Puelles et al., 2007) and ventral periventricular nucleus (Puelles et al., 2007), stratum cellullare internum and zona incerta, vasotocinergic fibers were also identified in the canary (Kiss et al., 1987). Unlike in blue tits, AVT+ fibers have been found in the thalamic anterior lateral nucleus and thalamic posterior dorsolateral nucleus, nucleus rotundus, and the ventral lateral geniculate nucleus in white-throated sparrows, zebra finches (Leung et al., 2009), quails and fowls (Panzica et al., 1988), and budgerigars (Fabris et al., 2004). To our knowledge, habenular vasotocinergic fibers have only been observed in the tits (present data), canaries (Kiss et al., 1987) and zebra finches (Voorhuis and De Kloet, 1992). Vasotocinergic fibers are also present in several mesencephalic and brainstem centers (optic tectum, midbrain central gray, intercollicular nucleus, lateral mesencephalic formation, ventral tegmental area, substantia nigra, locus coeruleus, raphe nuclei) both in tits and in other bird species (present data), (Kiss et al., 1987; Panzica et al., 1988, 1999, 2001; Voorhuis and De Kloet, 1992; Leung et al., 2009). The scarcity of AVT+ fibers observed in the locus coeruleus of tits is in contrast with their abundance in quails (see Figure 4M,N in Panzica et al., 2001). In pretectal nuclei, AVT+ fibers have been noticed in song birds (Panzica et al., 1999; Leung et al., 2009), but not in galliform species. Vasotocinergic fibers have been noted in the dorsal and ventral subcoeruleus nuclei, reticular pontine formation of quails and fowls (Panzica et al., 1988), and they were also evident in tits. Unlike in tits, AVT+ fibers were also observed in the nucleus of Edinger–Westphal (white-throated sparrow, Leung et al., 2009), the nucleus of the basal optic root [aka nucleus ectomammillaris—quail and fowl (Panzica et al., 1988, 2001), and the dorsal nucleus of the oculomotor nerve (white-throated sparrow and zebra finches, Leung et al., 2009).

### Distribution of VIP neurons and fibers and comparison with other avian species

VIP+ neurons are mainly grouped in the lateral septal organ and in the tuberal areas of the hypothalamus. These two groups are present in ring doves (Peczely and Kiss, 1988; Cloues et al., 1990; Kiyoshi et al., 1998), pigeons (Hof et al., 1991), collared doves (Den Boer-Visser and Dubbeldam, 2002), dark-eyed juncos (Deviche et al., 2000), bantam and Thai hen (Macnamee et al., 1986; Kosonsiriluk et al., 2008; Prakobsaeng et al., 2011), chicken (Esposito et al., 1993; Kuenzel and Blahser, 1994), Japanese quails (Yamada et al., 1982; Aste et al., 1995; Teruyama and Beck, 2001).

We did not observe other VIP+ neurons in the telencephalon or the diencephalon. This situation is similar to that observed in dark-eyed juncos (Deviche et al., 2000) and zebra finches (Bottjer and Alexander, 1995). In two songbirds (Song sparrow and starling), VIP+ neurons were observed in the arcopallium surrounding nucleus robustus and the caudal nidopallium (Ball et al., 1988), whereas such neurons were not identified in the tits and zebra finches (Bottjer and Alexander, 1995). Some neurons were immunostained for VIP+ in the lateral and medial striatum of zebra finches (Bottjer and Alexander, 1995). We did not observe stained neurons in these structures of tits.

Blue tits appear very similar in the staining of the hypothalamus to that found in zebra finches (Bottjer and Alexander, 1995) because the only subregion to contain VIP+ neurons was the arcuate nucleus. This is at variance with the situation in several other avian species where additional locations for VIP neurons have been reported: the medial and lateral hypothalamic area, anterior, supraoptic, suprachiasmatic, paraventricular, periventricular, submammillary and premammillary nuclei in doves, pigeons, Japanese quails and chicken (Peczely and Kiss, 1988; Cloues et al., 1990; Norgren and Silver, 1990; Hof et al., 1991; Esposito et al., 1993; Kuenzel and Blahser, 1994; Aste et al., 1995; Teruyama and Beck, 2001; Den Boer-Visser and Dubbeldam, 2002; Kosonsiriluk et al., 2008; Prakobsaeng et al., 2011).

No VIP+ neurons were observed in the thalamus of tit, chicken (Kuenzel and Blahser, 1994) or zebra finch (Bottjer and Alexander, 1995). In doves, scattered VIP+ neurons have been identified in the dorsal thalamus (Cloues et al., 1990; Den Boer-Visser and Dubbeldam, 2002). In the Thai hen, VIP+ neurons are present in the nucleus rotundus (Kosonsiriluk et al., 2008).

In the brainstem of the blue tit, scattered VIP+ neurons were found in the perirubral region, substantia nigra, ventral subcoeruleus nucleus and pontine reticular formation. Some scattered VIP+ neurons were also observed in the midbrain central gray, intercollicular nucleus, ventral tegmental area, interpeduncular nucleus, substantia nigra, optic tectum, locus coeruleus, ventral subcoeruleus, lateral paragigantocellular reticular nuclei of collared doves, Japanese quail, young chicken and adult hen and songbirds (zebra finch, song sparrow and starling) (Ball et al., 1988; Kuenzel and Blahser, 1994; Aste et al., 1995; Bottjer and Alexander, 1995; Den Boer-Visser and Dubbeldam, 2002; Kosonsiriluk et al., 2008).

VIP+ fibers and terminal fields are extensively distributed throughout the hypothalamus, essentially in the anterior nucleus, lateral hypothalamic area, paraventricular, periventricular and tuberal and infundibular nuclei and median eminence. Their occurrence is very similar in many other species [zebra finch (Bottjer and Alexander, 1995), pigeon (Hof et al., 1991; Den Boer-Visser and Dubbeldam, 2002), dove (Den Boer-Visser and Dubbeldam, 2002), quail (Yamada et al., 1982; Aste et al., 1995), Bantam and Thai hen (Macnamee et al., 1986; Kosonsiriluk et al., 2008), chicken (Esposito et al., 1993; Kuenzel and Blahser, 1994)].

Common to all bird species is the strong VIP immunostaining of fibers in the lateral septal areas [present data, pigeon (Peczely and Kiss, 1988; Hof et al., 1991; Kiyoshi et al., 1998), dove (Den Boer-Visser and Dubbeldam, 2002), Bantam and Thai hen (Macnamee et al., 1986; Kosonsiriluk et al., 2008), chicken (Kuenzel and Blahser, 1994), quail (Yamada et al., 1982; Aste et al., 1995, 1997), zebra finches (Bottjer and Alexander, 1995), estrildid and emberizid species (Goodson et al., 2004)]. In the tits, the bed nucleus of the stria terminalis and the nucleus of the pallial commissure contain an important contingent of VIP immunoreactive fibers which has been observed also in the zebra finch (Bottjer and Alexander, 1995) and quail (Aste et al., 1995), but not in the pigeon, dove, and chicken (Hof et al., 1991; Kuenzel and Blahser, 1994; Den Boer-Visser and Dubbeldam, 2002).

In the tit, VIP+ fibers are found all over the preoptic area, including the medial preoptic nucleus, as in zebra finches (Bottjer and Alexander, 1995). VIP+ fibers occur in the anterior preoptic nucleus of the pigeon (Hof et al., 1991), the preoptic area of the quail excepting the medial preoptic nucleus (Aste et al., 1995) and in the ventral preoptic region of the chicken (Kuenzel and Blahser, 1994). It has to be noted that all the above mentioned studies were done on males. Thus, intersexual differences cannot be accounted for any observed differences in labeled structures, while these may well be related to functional changes in reproductive status.

VIP+ fibers are also observed in other telencephalic areas such as the area corticoidea dorsolateralis (tits, present data; pigeon, Hof et al., 1991), medial hyperpallium apicale (tits; zebra finch, Bottjer and Alexander, 1995), hyperpallium densocellulare (tits; pigeon, Hof et al., 1991), hippocampal area [tits; collared dove (Den Boer-Visser and Dubbeldam, 2002), homing pigeon (Erichsen et al., 1991)], arcopallium [tits; zebra finch (Bottjer and Alexander, 1995), pigeon (Hof et al., 1991), chicken (Kuenzel and Blahser, 1994), quail (Yamada et al., 1982; Aste et al., 1995)] including the amygdaloid nucleus taeniae (tits; quail, Aste et al., 1995), olfactory tubercle [tits; pigeon (Hof et al., 1991), quail (Aste et al., 1995)], nucleus accumbens [tits; pigeon (Hof et al., 1991), quail (Yamada et al., 1982), Thai hen (Kosonsiriluk et al., 2008)], along the medial wall of the medial striatum [pigeon (Hof et al., 1991), quail (Aste et al., 1995), Thai hen (Kosonsiriluk et al., 2008)], medial septal nuclei [tits; pigeon (Hof et al., 1991), Thai hen (Kosonsiriluk et al., 2008)], ventral pallidum [tits; chicken (Kuenzel and Blahser, 1994), quail (Aste et al., 1995)], the piriform cortex in chicken (Kuenzel and Blahser, 1994). It should be noted that in the zebra finches, song sparrow and starling, VIP+ fibers and terminals are abundant in the medial magnocellular nucleus of the anterior nidopallium, mesopallial higher vocal center and the arcopallial robust nucleus (Ball et al., 1988, 1995; Bottjer and Alexander, 1995). In the tit we were unable to identify the medial magnocellular nucleus of the anterior nidopallium, and few fibers were present in the caudal mesopallium. No fibers were identified in the arcopallial robust nucleus proper but a few were seen around it, as in zebra finches (Bottjer and Alexander, 1995). Notably, the area previously defined as hyperpallium densocellulare has now been termed mesopallium dorsale by Jarvis et al. (2013). According to the 3D-reconstruction of these authors, the medial mesopallial region is likely to be connected to the higher vocal center. Therefore, the VIP+ fibers observed in these two areas may in fact belong to the same functional unit.

In the thalamus, subthalamus and epithalamus, VIP+ fibers are more commonly found in the thalamic dorsomedial and dorsolateral nuclei [present data, pigeon (Hof et al., 1991), quail (Aste et al., 1995)]. They are also found in the zona incerta (present data, collared dove, Den Boer-Visser and Dubbeldam, 2002), medial habenular nucleus in chicken (Kuenzel and Blahser, 1994), quail (Aste et al., 1995), lateral habenular nucleus in zebra finches (Bottjer and Alexander, 1995).

In the lower brainstem of tits, VIP+ fibers and terminals are present mainly in the midbrain central gray, ventral tegmental area, intercollicular nucleus, optic tectum and pontine reticular formation, less frequently in the pretectal nuclei, mesencephalic reticular formation, substantia nigra, locus coeruleus, subcoeruleus nuclei, raphe structures. This distribution is fairly similar to that observed in other species: optic tectum, midbrain central gray, intercollicular nucleus mesencephalic reticular formation, in and around the posterior and tectal commissures, ventral tegmental area, interpeduncular nucleus, substantia nigra, dorsal tegmentum raphe area, locus coeruleus, nucleus subcoeruleus, the 7th, 9th, and 10th cranial nerve nuclei and surroundings, pontine reticular formation, fasciculus longitudinalis medialis, lemniscus lateralis, parabrachial area, motor nucleus of the trigeminal nerve, subtrigeminal reticular nucleus, caudal part of the plexus of Horsley, ventral part of the medial vestibular nucleus and nucleus of the solitary tract and in pigeons, collared doves, quails, chicken, Thai hen and zebra finches (Hof et al., 1991; Kuenzel and Blahser, 1994; Aste et al., 1995; Bottjer and Alexander, 1995; Den Boer-Visser and Dubbeldam, 2002; Kosonsiriluk et al., 2008).

### Phylogenetic considerations

The following comparative analysis was focused on sauropsids and mammals.

#### Vasotocinergic/vasopressinergic systems

The vasotocinergic system appears highly conserved in the vertebrates. The vasotocinergic hypothalamo-hypophyseal system is well developed in lizards (Goossens et al., 1979; Bons, 1983; Stoll and Voorn, 1985; Thepen et al., 1987; Propper et al., 1992; Bennis et al., 1995; Barka-Dahane et al., 2010), snakes (Fernandez-Llebrez et al., 1988; Silveira et al., 2002), turtles (Fernandez-Llebrez et al., 1988). It is to be noted that in lizards, as in birds, there are several accessory clusters of AVT+ neurons between the supraoptic and paraventricular nuclei (Goossens et al., 1979; Stoll and Voorn, 1985; Thepen et al., 1987; Propper et al., 1992; Barka-Dahane et al., 2010).

Extrahypothalamic AVT+ neurons are present in two sites: the bed nucleus of the stria terminalis and the caudal rhombencephalon (rhombencephalic inferior reticular nucleus) in geckos (Stoll and Voorn, 1985; Thepen et al., 1987), but not in other lizard species (Propper et al., 1992; Bennis et al., 1995). AVT+ fibers have been observed in the preoptic area, ventral telencephalon (diagonal band of Broca, nucleus accumbens, and tuberculum olfactorium), nucleus sphericus, bed nucleus of stria terminalis, anterior septal nucleus and lateral septum, dorsolateral and ventrolateral thalamic nuclei, mesencephalic tectum, periaqueductal gray, along the fourth ventricle, around the descending nucleus of the trigeminal nerve, and rhombencephalic tegmentum (possibly substantia nigra and locus coeruleus equivalents) (Stoll and Voorn, 1985; Thepen et al., 1987; Propper et al., 1992; Bennis et al., 1995; Barka-Dahane et al., 2010). In the lateral septum and the ventral pole of the nucleus sphericus, AVT+ innervation is sexually dimorphic (Stoll and Voorn, 1985; Propper et al., 1992) except in chameleon (Bennis et al., 1995). In snakes and turtles, AVT+ neurons were observed in the dorsolateral aggregation, dorsal to the lateral forebrain bundle and in the recessus infundibularis nucleus, but none were identified in the bed nucleus of the stria terminalis and the rhombencephalic inferior reticular nucleus (Fernandez-Llebrez et al., 1988; Silveira et al., 2002). AVT+ fibers are absent from septal areas and rare in the mesencephalon and rhombencephalon (Silveira et al., 2002).

In mammals, vasotocin is replaced by vasopressin. Notwithstanding the rather long list of brain regions involved, a general conclusion can be drawn according to which the distribution of vasopressin-containing structures (both perikarya and fibers) is rather similar to the distribution of AVT in most avian species studied. The hypothalamo-hypophyseal vasopressinergic system of mammals is particularly similar to that of birds: vasopressinergic magnocellular neurons are found in the supraoptic and paraventricular nuclei and some accessory small nuclei (Palkovits, 1984; De Vries et al., 1985; Caffe et al., 1989; Hermes et al., 1990; Van Eerdenburg et al., 1992; Luo et al., 1995; Wang et al., 1996, 1997; Rosen et al., 2006, 2007; Rood and De Vries, 2011; Otero-Garcia et al., 2014) for review (Moore and Lowry, 1998; De Vries and Miller, 1999; Caldwell and Young, 2006).

Apart from magnocellular hypothalamic nuclei, by far the best known part of vasopressin distribution, the peptide is also present in other hypothalamic and extrahypothalamic nuclei. The first group comprises the suprachiasmatic nucleus [excepting tupaia (Luo et al., 1995) or the naked mole rat (Rosen et al., 2007)], the lateral hypothalamic and periventricular area, dorsomedial hypothalamic nucleus, posterodorsal hypothalamic area, dorsal capsule of the ventromedial hypothalamic nucleus and the arcuate nucleus and its surroundings (De Vries et al., 1985; Caffe et al., 1989; Van Eerdenburg et al., 1992; Luo et al., 1995; Wang et al., 1996, 1997; Ibata et al., 1999; Rosen et al., 2006, 2007; Rood and De Vries, 2011) for review (Moore and Lowry, 1998; De Vries and Miller, 1999; Caldwell and Young, 2006). In most mammalian species, extrahypothalamic vasopressinergic neurons have been observed in the bed nucleus of stria terminalis and the medial nucleus of amygdala (De Vries et al., 1985; Fliers et al., 1986; Caffe et al., 1989; Hermes et al., 1990; Ferris et al., 1995; Wang et al., 1997; Rosen et al., 2007; Rood and De Vries, 2011; Otero-Garcia et al., 2014) for review (Moore and Lowry, 1998; De Vries and Miller, 1999; Caldwell and Young, 2006). Less frequent sites of occurrence, inconsistent among mammalian species, are the diagonal band of Broca, lateral and medial septal areas, basal nucleus of Meynert, preoptic area, the internal part of the globus pallidus, deep mesencephalic nucleus, locus coeruleus and its surroundings, nucleus subcoeruleus, raphe region, periolivary region, nucleus of the solitary tract, (De Vries et al., 1985; Caffe et al., 1989; Wang et al., 1996; Rosen et al., 2007; Rood and De Vries, 2011) for review (Moore and Lowry, 1998; Caldwell and Young, 2006), even as far as the cerebellum (Rosen et al., 2007) and spinal cord (Caffe et al., 1989).

Concerning vasopressin-containing fibers, apart from their well-known presence in the supraoptic and paraventricular nuclei, the hypothalamo-hypophyseal tract and the medial eminence (not discussed in detail), the most notable sites in the hypothalamus largely correspond to those listed above with respect to perikaryal distribution, with notable additions: anterior hypothalamic area, premammillary and supramammillary nuclei, parastriatal nucleus (Palkovits, 1984; De Vries et al., 1985; Caffe et al., 1989; Hermes et al., 1990; Lantos et al., 1995; Luo et al., 1995; Wang et al., 1996, 1997; Moore and Lowry, 1998; Rosen et al., 2006, 2007; Rood and De Vries, 2011; Otero-Garcia et al., 2014).

Extrahypothalamic vasopressinergic fibers are present in many areas in the entire brain. Diencephalic centers include medial thalamic nuclei, the area around the fasciculus retroflexus, zona incerta and lateral habenular nucleus (Palkovits, 1984; De Vries et al., 1985; Buijs et al., 1986; Caffe et al., 1989; Hermes et al., 1990; Wang et al., 1996; Caldwell and Young, 2006; Rosen et al., 2006, 2007; Rood and De Vries, 2011; Otero-Garcia et al., 2014). In the telencephalon, vasopressinergic fibers are regularly found in the bed nucleus of stria terminalis, lateral septum, preoptic region (mainly medial and periventricular preoptic nuclei) and the amygdaloid nuclei (essentially its medial nucleus) (Palkovits, 1984; De Vries et al., 1985; Buijs et al., 1986; Fliers et al., 1986; Caffe et al., 1989; Hermes et al., 1990; Wang et al., 1996, 1997; Caldwell and Young, 2006; Rosen et al., 2006, 2007; Rood and De Vries, 2011; Otero-Garcia et al., 2014). However, in the macaque, vasopressinergic fibers have not been observed in the lateral septum (Caffe et al., 1989; Wang et al., 1997). Besides, in a few species the medial septum (Rosen et al., 2006, 2007), including its transitional zone (Palkovits, 1984; De Vries et al., 1985), septofimbrial nucleus (De Vries et al., 1985), dorsal septum (Palkovits, 1984) and the bed nucleus of the anterior commissure (Fliers et al., 1986) also contain peptidergic fibers. Furthermore, vasopressinergic fibers were encountered in the olfactory bulb and tubercle, nucleus of the diagonal band of Broca, cortex (entorhinal, cingulate, prefrontal, piriform), hippocampal formation, ventral pallidum, nucleus accumbens, basal nucleus of Meynert and endopiriform nucleus (Palkovits, 1984; De Vries et al., 1985; Fliers et al., 1986; Caffe et al., 1989; Hermes et al., 1990; Wang et al., 1996, 1997; Caldwell and Young, 2006; Rosen et al., 2006, 2007; Rood and De Vries, 2011; Otero-Garcia et al., 2014). In the midbrain and the pons, vasopressinergic fibers were observed in central gray, substantia nigra, ventral tegmental area, raphe nuclei, locus coeruleus and subcoeruleus, pontine tegmental area, parabrachial nuclei, nucleus of the solitary tract, reticular formation, and several cranial nerve nuclei (Palkovits, 1984; De Vries et al., 1985; Caffe et al., 1989; Hermes et al., 1990; Maley, 1996; Caldwell and Young, 2006; Rosen et al., 2007; Rood and De Vries, 2011; Otero-Garcia et al., 2014). In addition, the presence of vasopressin fibers is notable in some regions of the auditory pathway (lateral superior olive region, nucleus lemnisci lateralis, Palkovits, 1984; Hermes et al., 1990). Fibers were observed in some subependymal regions such as the organum vasculosum laminae terminalis, subfornical organ and subcommissural organ and the area postrema (Palkovits, 1984; De Vries et al., 1985; Hermes et al., 1990; Rosen et al., 2006, 2007; Rood and De Vries, 2011).

As in birds, several brain areas including the lateral septum, bed nucleus of the stria terminalis, amygdaloid areas, ventral tegmental areas are sexually dimorphic in the distribution of vasopressinergic fibers (De Vries et al., 1985; Buijs et al., 1986; Crenshaw et al., 1992; Wang et al., 1996, 1997; Caldwell and Young, 2006; Rosen et al., 2007; De Vries, 2008), although in some species no sexual differences have been noted [hamster (Caldwell and Young, 2006; Bolborea et al., 2010), human (Fliers et al., 1986)].

Overall, there seem to be marked similarities between the neuroanatomical organization of AVT/vasopressin containing nuclei and fiber tracts in the different functional systems of birds and mammals.

#### VIP system

In reptiles, data on VIP+ distribution in the brain is rare and fragmentary. In all reptile families (chelonian, lacertilian, ophidian, crocodilian), VIP immunoreactivity is present in cerebrospinal fluid-contacting neurons of the lateral septal organ at the level of the nucleus accumbens/lateral septum of lizards (Petko and Ihionvien, 1989; Hirunagi et al., 1993; Grace et al., 1996). VIP+ neurons have also been identified in the suprachiasmatic nucleus and in a mesencephalic region ventrolateral to the medial longitudinal fascicle in lizards (Petko and Ihionvien, 1989; Magnone et al., 2003), and the supramammillary/lateral hypothalamic region in turtles (Reiner, 1991). VIP+ fibers distribution is more widespread including the dorsal cortex and pallial thickening of the turtles (Reiner, 1991), basket like structure around neurons in the lateral septum and the nucleus accumbens (Hirunagi et al., 1993), telencephalic and hypothalamic periventricular gray matter, hypothalamic paraventricular nucleus, infundibulum, the lateral part of the torus semicircularis and mesencephalic area ventrolateral to the medial longitudinal fascicle, the rhombencephalic subventricular and dorsolateral part of the tegmentum, parabrachial nucleus area, and central gray matter and in the medulla oblongata, subependimal zone, solitary nucleus and dorsal vagal motor nucleus areas as well as the descending tract and nucleus of the trigeminal nerve (Petko and Ihionvien, 1989).

The distribution of VIP+ neurons across the brain is very similar between mammalian species, although some minor differences do exist. In the telencephalon, they have been identified in the cortex, caudate-putamen, claustrum, olfactory bulb, olfactory tubercle, hippocampus, bed nucleus of the stria terminalis, interstitial nucleus of the stria terminalis, amygdala, septum, medial preoptic area (Fuxe et al., 1977; Lorén et al., 1979; Roberts et al., 1980; Sims et al., 1980; Obata-Tsuto et al., 1983; Palkovits, 1984; Ramon Y Cajal-Agueras et al., 1986; Antonopoulos et al., 1987; Laemle and Cotter, 1988; Ibata et al., 1999). In the diencephalon, most VIP+ neurons are found in the suprachiasmatic nucleus (Lorén et al., 1979; Roberts et al., 1980; Sims et al., 1980; Card et al., 1981; Obata-Tsuto et al., 1983; Palkovits, 1984; Ibata et al., 1999). Some VIP+ neurons are also present in supraoptic, paraventricular and, periventricular nuclei, premammillary region and arcuate nucleus (Roberts et al., 1980; Sims et al., 1980; Card et al., 1981; Obata-Tsuto et al., 1983; Palkovits, 1984; Antonopoulos et al., 1987; Simerly and Swanson, 1987; Laemle and Cotter, 1988; Larsen and Mikkelsen, 1992; Lantos et al., 1995). Further VIP+ neurons were observed in the mesencephalon (central gray, superior collicle, and raphe nuclei), in the subependymal neuropil of the ventromedial and ventral periaqueductal gray, medulla oblongata (nucleus tractus solitarii) and spinal cord, nucleus interfascicularis (Lorén et al., 1979; Sims et al., 1980; Moss and Basbaum, 1983; Obata-Tsuto et al., 1983; Palkovits, 1984; Antonopoulos et al., 1987; Laemle and Cotter, 1988; Maley, 1996; Ahnaou et al., 2006).

VIP+ fibers are found not only in the above mentioned areas, but are widespread in many other structures. In the telencephalon, they are found in the nucleus accumbens, head of the caudate nucleus, globus pallidus, substantia innominata, preoptic area, nucleus tractus diagonalis, rostral medial forebrain bundle, central nucleus of the amygdala (Lorén et al., 1979; Roberts et al., 1980; Sims et al., 1980; Palkovits, 1984). In the diencephalon VIP+ fibers innervate the thalamus, hypothalamus, subthalamic zona incerta, medial habenula (Fuxe et al., 1977; Lorén et al., 1979; Roberts et al., 1980; Sims et al., 1980; Palkovits, 1984; Simerly and Swanson, 1987; Lantos et al., 1995). In the midbrain and the pons, VIP+ fibers are present in the red nucleus, substantia nigra, ventral tegmental area, interpeduncular nucleus, raphe nuclei, inferior colliculus, reticular and dorsal tegmental nuclei, parabrachial and lateral lemnicus nuclei and oliva superior, medulla oblongata (motor facial, lateral reticular, ambiguous, gracile, and paramedian nuclei, inferior olive, area postrema) and spinal cord (Lorén et al., 1979; Sims et al., 1980; Moss and Basbaum, 1983; Obata-Tsuto et al., 1983; Palkovits, 1984; Maley, 1996; Ahnaou et al., 2006). In summary, four major VIP systems are present in mammals: (1) an intracortical system; (2) a system centered on the amygdala and the bed nucleus of the stria terminalis, also connecting with the nucleus accumbens, septal areas and the hypothalamus; (3) a pathway originating from the suprachiasmatic nucleus, connected with hypothalamic and thalamic areas; (4) a pathway originating in the central gray of the midbrain (Lorén et al., 1979; Sims et al., 1980; Rosténe, 1984).

Immunoreactivity to VIP in the form of perikarya and fibers were certainly detected in regions potentially corresponding to all of the above mentioned systems in the tit species studied here. The only exception could be a conspicuous lack of labeling in the suprachiasmatic nucleus.

### Functional consideration

For the present study, blue tits observed were caught during the parental stage of the reproductive cycle, when both males and females feed and care for the hatchlings. Such parental behavior requires cooperation between males and females and the existence of social bond between the parents as well as between parents and offspring. In all other studies on AVT distribution, the bird species used as models either had a different mating system/social organization (Aste et al., 1995; Panzica et al., 1997; Goodson and Kingsbury, 2011; Lynn, 2015), or they were in different reproductive stages (Jurkevich and Grossmann, 2003; Xie et al., 2011). Not surprisingly, the main differences between the AVT distribution in the tit and that observed in other avian species occurred in those brain regions that are responsible for various aspects of social and reproductive behaviors. Nucleus accumbens, ventral pallidum and the medial striatum are involved, among other functions, in social reward and motivation (Zheng et al., 2013). The lateral septum, which is an important target of steroids (Panzica et al., 1997) and contains GnRH neurons in its rostral portion, close to the preoptic area (Kuenzel and Blahser, 1991), belongs to a brain network regulating social behavior in many vertebrates (Goodson and Kingsbury, 2013), In the blue tit, the apparent lack of sexual dimorphism in the preoptic area, a region regulating copulatory behavior (Balthazart and Ball, 2007), could be linked to the fact that this species is monogamous and biparental, both male and female behavior and physiology being rather similar during parental care. Similarly, in dorsal hypothalamus, which is involved in the regulation of aggression (Goodson and Kabelik, 2009), the weaker AVT immunoreactivity in the blue tit as compared to other species, is consistent with reduced aggressive behavior observed in blue tits at the onset of egglaying (Kempenaers, 1995). It should be noted that there are no interspecific differences in the medial hypothalamic AVT+ cell groups, which are more related to water and salt homeostasis, rather than behavioral functions (Seth et al., 2004). AVT staining proved to be weak and without sexual differences in the locus coeruleus of the blue tit. This brain region can also be influenced by social behavior (Lynch et al., 2012), however its role in avian social behavior is hardly studied. Habenula, where AVT is more abundant in tits is also possibly part of the extended social brain network (Goodson and Kingsbury, 2013). Little is known about the functions of AVT in the habenula and the pretectum, another site where difference occurs between galliforms and songbirds.

The weak labeling of VIP in the telencephalon of blue tits, especially in the song system, can also be ascribed to interspecific differences in song learning, but also to the fact that courtship songs are less frequent during parental care (Hill et al., 2005). VIP is the releaser of prolactine, the hormone responsible for physiological and behavioral changes during parental care and also a neuromodulator, itself associated with parental behavior (Badyaev and Duckworth, 2005; Kingsbury et al., 2015). Intense expression of VIP in the bed nucleus of stria terminalis is in harmony with the parenting stage of blue tits in this study.

## General conclusion

In summary, this is the first comprehensive description of the vasotocinergic and VIP-ergic systems in the brain of blue tit. The overall distribution of vasotocine and VIP- immunoreactive neurons, fibers and terminal fields remain conservative not only within songbirds, but throughout the evolutionary history of vertebrates. It seems very likely that neuropeptide expression levels are subject to changes during seasonal, behavioral and physiological transitions, therefore, in functional anatomical studies, both the differences in reproductive stages within a species, and interspecific differences of mating systems represent important factors.

## Author contributions

GZ did the entire field work and participated in tissue processing and information technology. CM was in charge of histological procedures, immunocytochemistry, the collection of microscopic observations and, together with AC, the analysis of results. CM, GZ, AC, and TS contributed to the final format, style and English of the manuscript.

### Conflict of interest statement

The authors declare that the research was conducted in the absence of any commercial or financial relationships that could be construed as a potential conflict of interest.

## Abbreviations

AVT: Vasotocine
AVT+: Vasotocine immunoreactive
VIP: Vasoactive intestinal peptide
VIP+: Vasoactive intestinal peptide immunoreactive.

## References

[B1] Ahnaou A. Yon L. Arluison M. Vaudry H. Hannibal J. Hamon M.. (2006). Immunocytochemical distribution of VIP and PACAP in the rat brain stem: implications for REM sleep physiology. Ann. N.Y. Acad. Sci. 1070, 135–142. 10.1196/annals.1317.095 16888155

[B2] Alcock J. (2009). Animal Behavior: An Evolutionary Approach. Sunderland: Sinauer Associates.

[B3] Antonopoulos J. Papadopoulos G. C. Karamanlidis A. N. Parnavelas J. G. Dinopoulos A. Michaloudi H. (1987). VIP- and CCK-like-immunoreactive neurons in the hedgehog (*Erinaceus europaeus*) and sheep (*Ovis aries*) brain. J. Comp. Neurol. 263, 290–307. 10.1002/cne.902630211 3312309

[B4] Aplin L. M. Sheldon B. C. Morand-Ferron J. (2013). Milk bottles revisited: social learning and individual variation in the blue tit, *Cyanistes caeruleus*. Anim. Behav. 85, 1225–1232. 10.1016/j.anbehav.2013.03.009

[B5] Aste N. Balthazart J. Absil P. Grossmann R. Mulhbauer E. Viglietti-Panzica C.. (1998). Anatomical and neurochemical definition of the nucleus of the stria terminalis in Japanese quail (*Coturnix japonica*). J. Comp. Neurol. 396, 141–157. 9634138

[B6] Aste N. Sakamoto E. Kagami M. Saito N. (2013). Vasotocin mRNA expression is sensitive to testosterone and oestradiol in the bed nucleus of the stria terminalis in female Japanese quail. J. Neuroendocrinol. 25, 811–825. 10.1111/jne.12076 23841557

[B7] Aste N. Viglietti-Panzica C. Balthazart J. (1997). Testosterone modulation of peptidergic pathways in the septo-preoptic region of male Japanese Quail. Poult. Avian Biol. Rev. 8, 77–93.

[B8] Aste N. Viglietti-Panzica C. Fasolo A. Panzica G. C. (1995). Mapping of neurochemical markers in quail central nervous system: VIP- and SP-like immunoreactivity. J. Chem. Neuroanat. 8, 87–102. 10.1016/0891-0618(94)00031-N 7541207

[B9] Badyaev A. Duckworth R. (2005). Evolution of plasticity in hormonally-integrated parental tactics: an example with the house finch, in Functional Avian Endocrinology, eds Dawson A. Sharp P. J. (New Delhi: Narosa Publishing House), 375–386.

[B10] Ball G. F. Absil P. Balthazart J. (1995). Assessment of volumetric sex differences in the song control nuclei HVC and RA in zebra finches by immunocytochemistry for methionine enkephalin and vasoactive intestinal polypeptide. Brain Res. 699, 83–96. 10.1016/0006-8993(95)00875-Q 8616616

[B11] Ball G. F. Faris P. L. Hartman B. K. Wingfield J. C. (1988). Immunohistochemical localization of neuropeptides in the vocal control regions of two songbird species. J. Comp. Neurol. 268, 171–180. 10.1002/cne.902680204 2452178

[B12] Balthazart J. Absil P. Viglietti-Panzica C. Panzica G. C. (1997). Vasotocinergic innervation of areas containing aromatase-immunoreactive cells in the quail forebrain. J. Neurobiol. 33, 45–60. 9212069

[B13] Balthazart J. Ball G. F. (2007). Topography in the preoptic region: differential regulation of appetitive and consummatory male sexual behaviors. Front. Neuroendocrinol. 28, 161–178. 10.1016/j.yfrne.2007.05.003 17624413 PMC2100381

[B14] Barka-Dahane Z. Bendjelloul M. Estabel J. Exbrayat J. M. (2010). The distribution of vasotocin and mesotocin immunoreactivity in the hypothalamic magnocellular neurosecretory nuclei of the Saharan herbivorous lizard, Uromastix acanthinurus Bell, 1825 (Sauria-Agamidae). Histol. Histopathol. 25, 159–175. Available online at: http://www.hh.um.es/Abstracts/Vol_25/25_2/25_2_159.htm 20017103 10.14670/HH-25.159

[B15] Bennett P. Owens I. (2002). Evolutionary Ecology of Birds - Life Histories, Mating Systems, and Extinction. Oxford: Oxford University Press.

[B16] Bennis M. Tramu A. M. Reperant J. (1995). Vasopressin- and oxytocin-like systems in the chameleon brain. J. Hirnforsch. 36, 445–450. 8568214

[B17] Berk M. L. Reaves T. A. Jr. Hayward J. N. Finkelstein J. A. (1982). The localization of vasotocin and neurophysin neurons in the diencephalon of the pigeon, *Columba livia*. J. Comp. Neurol. 204, 392–406. 10.1002/cne.902040410 7061740

[B18] Bolborea M. Ansel L. Weinert D. Steinlechner S. Pevet P. Klosen P. (2010). The bed nucleus of the stria terminalis in the Syrian hamster (*Mesocricetus auratus*): absence of vasopressin expression in standard and wild-derived hamsters and galanin regulation by seasonal changes in circulating sex steroids. Neuroscience 165, 819–830. 10.1016/j.neuroscience.2009.11.006 19909796

[B19] Bons N. (1980). The topography of mesotocin and vasotocin systems in the brain of the domestic mallard and Japanese quail: immunocytochemical identification. Cell Tissue Res. 213, 37–51. 10.1007/BF00236919 7459995

[B20] Bons N. (1983). Immunocytochemical identification of the mesotocin- and vasotocin-producing systems in the brain of temperate and desert lizard species and their modifications by cold exposure. Gen. Comp. Endocrinol. 52, 56–66. 10.1016/0016-6480(83)90158-2 6628979

[B21] Bottjer S. W. Alexander G. (1995). Localization of met-enkephalin and vasoactive intestinal polypeptide in the brains of male zebra finches. Brain Behav. Evol. 45, 153–177. 10.1159/000113547 7796094

[B22] Buijs R. M. Pevet P. Masson-Pevet M. Pool C. W. De Vries G. J. Canguilhem B.. (1986). Seasonal variation in vasopressin innervation in the brain of the European hamster (*Cricetus cricetus*). Brain Res. 371, 193–196. 10.1016/0006-8993(86)90829-2 3708343

[B23] Caffe A. R. Van Ryen P. C. Van Der Woude T. P. Van Leeuwen F. W. (1989). Vasopressin and oxytocin systems in the brain and upper spinal cord of *Macaca fascicularis*. J. Comp. Neurol. 287, 302–325. 10.1002/cne.902870304 2778107

[B24] Caldwell H. K. Young W. S. III. (2006). Oxytocin and vasopressin: genetics and behavioral implications, in Handbook of Neurochemistry and Molecular Neurobiology, eds Lajtha A. Lim R. (New York, NY: Springer), 573–607.

[B25] Card J. P. Brecha N. Karten H. J. Moore R. Y. (1981). Immunocytochemical localization of vasoactive intestinal polypeptide-containing cells and processes in the suprachiasmatic nucleus of the rat: light and electron microscopic analysis. J. Neurosci. 1, 1289–1303. 7031198 10.1523/JNEUROSCI.01-11-01289.1981 PMC6564223

[B26] Chaiseha Y. Youngren O. M. El Halawani M. E. (2004). Expression of vasoactive intestinal peptide receptor messenger RNA in the hypothalamus and pituitary throughout the turkey reproductive cycle. Biol. Reprod. 70, 593–599. 10.1095/biolreprod.103.022715 14568918

[B27] Cloues R. Ramos C. Silver R. (1990). Vasoactive intestinal polypeptide-like immunoreactivity during reproduction in doves: influence of experience and number of offspring. Horm. Behav. 24, 215–231. 10.1016/0018-506X(90)90006-J 2194925

[B28] Crenshaw B. J. De Vries G. J. Yahr P. (1992). Vasopressin innervation of sexually dimorphic structures of the gerbil forebrain under various hormonal conditions. J. Comp. Neurol. 322, 589–598. 10.1002/cne.903220412 1401252

[B29] Davies N. B. (1992). Dunnock Behaviour and Social Evolution. Oxford: Oxford University Press.

[B30] De Vries G. J. (2008). Sex differences in vasopressin and oxytocin innervation of the brain, in Progress in Brain Research, eds Inga D. N. Rainer L. (Amsterdam: Elsevier), 17–27. 10.1016/S0079-6123(08)00402-0 18655868

[B31] De Vries G. J. Buijs R. M. Van Leeuwen F. W. Caffe A. R. Swaab D. F. (1985). The vasopressinergic innervation of the brain in normal and castrated rats. J. Comp. Neurol. 233, 236–254. 10.1002/cne.902330206 3882778

[B32] De Vries G. J. Miller M. A. (1999). Chapter 1.1 Anatomy and function of extrahypothalamic vasopressin systems in the brain. Prog. Brain Res. 119, 3–20. 10074777 10.1016/s0079-6123(08)61558-7

[B33] Den Boer-Visser A. M. Dubbeldam J. L. (2002). The distribution of dopamine, substance P, vasoactive intestinal polypeptide and neuropeptide Y immunoreactivity in the brain of the collared dove, *Streptopelia decaocto*. J. Chem. Neuroanat. 23, 1–27. 10.1016/S0891-0618(01)00138-7 11756007

[B34] Deviche P. Saldanha C. J. Silver R. (2000). Changes in brain gonadotropin-releasing hormone- and vasoactive intestinal polypeptide-like immunoreactivity accompanying reestablishment of photosensitivity in male dark-eyed juncos (*Junco hyemalis*). Gen. Comp. Endocrinol. 117, 8–19. 10.1006/gcen.1999.7361 10620420 PMC3266068

[B35] Dickens M. Hartley I. R. (2007). Differences in parental food allocation rules: evidence for sexual conflict in the blue tit? Behav. Ecol. 18, 674–679. 10.1093/beheco/arm029

[B36] Erichsen J. T. Bingman V. P. Krebs J. R. (1991). The distribution of neuropeptides in the dorsomedial telencephalon of the pigeon (*Columba livia*): a basis for regional subdivisions. J. Comp. Neurol. 314, 478–492. 10.1002/cne.903140306 1726107

[B37] Esposito V. De Girolamo P. Gargiulo G. (1993). Immunoreactivity to vasoactive intestinal polypeptide (VIP) in the hypothalamus of the domestic fowl, *Gallus domesticus*. Neuropeptides 25, 83–90. 10.1016/0143-4179(93)90086-P 8413861

[B38] Fabris C. Ballarin C. Massa R. Granato A. Fabiani O. Panzica G. C.. (2004). The vasotocinergic system in the hypothalamus and limbic region of the budgerigar (*Melopsittacus undulatus*). Eur. J. Histochem. 48, 367–372. 10.4081/909 15718202

[B39] Feeney W. E. Medina I. Somveille M. Heinsohn R. Hall M. L. Mulder R. A.. (2013). Brood parasitism and the evolution of cooperative breeding in birds. Science 342, 1506–1508. 10.1126/science.1240039 24357317

[B40] Fernandez-Llebrez P. Perez J. Nadales A. E. Cifuentes M. Grondona J. M. Mancera J. M.. (1988). Immunocytochemical study of the hypothalamic magnocellular neurosecretory nuclei of the snake Natrix maura and the turtle *Mauremys caspica*. Cell Tissue Res. 253, 435–445. 10.1007/BF00222301 3409295

[B41] Ferris C. F. Delville Y. Miller M. A. Dorsa D. M. De Vries G. J. (1995). Distribution of small vasopressinergic neurons in golden hamsters. J. Comp. Neurol. 360, 589–598. 10.1002/cne.903600404 8801251

[B42] Fliers E. Guldenaar S. E. Van De Wal N. Swaab D. F. (1986). Extrahypothalamic vasopressin and oxytocin in the human brain; presence of vasopressin cells in the bed nucleus of the stria terminalis. Brain Res. 375, 363–367. 10.1016/0006-8993(86)90759-6 3524745

[B43] Foerster K. Kempenaers B. (2004). Experimentally elevated plasma levels of testosterone do not increase male reproductive success in blue tits. Behav. Ecol. Sociobiol. 56, 482–490. 10.1007/s00265-004-0809-2

[B44] Fuxe K. Hokfelt T. Said S. I. Mutt V. (1977). Vasoactive intestinal polypeptide and the nervous system: immunohistochemical evidence for localization in central and peripheral neurons, particularly intracortical neurons of the cerebral cortex. Neurosci. Lett. 5, 241–246. 10.1016/0304-3940(77)90073-8 19605001

[B45] Goodson J. L. (1998a). Territorial aggression and dawn song are modulated by septal vasotocin and vasoactive intestinal polypeptide in male field sparrows (*Spizella pusilla*). Horm. Behav. 34, 67–77. 10.1006/hbeh.1998.1467 9735230

[B46] Goodson J. L. (1998b). Vasotocin and vasoactive intestinal polypeptide modulate aggression in a territorial songbird, the violet-eared waxbill (Estrildidae: *Uraeginthus granatina*). Gen. Comp. Endocrinol. 111, 233–244. 10.1006/gcen.1998.7112 9679095

[B47] Goodson J. L. (2008). Nonapeptides and the evolutionary patterning of sociality, in Progress in Brain Research, eds Inga D. N. Rainer L. (Amsterdam: Elsevier), 3–15. 10.1016/S0079-6123(08)00401-9 PMC2570786 18655867

[B48] Goodson J. L. Kabelik D. (2009). Dynamic limbic networks and social diversity in vertebrates: from neural context to neuromodulatory patterning. Front. Neuroendocrinol. 30, 429–441. 10.1016/j.yfrne.2009.05.007 19520105 PMC2763925

[B49] Goodson J. L. Kelly A. M. Kingsbury M. A. (2012a). Evolving nonapeptide mechanisms of gregariousness and social diversity in birds. Horm. Behav. 61, 239–250. 10.1016/j.yhbeh.2012.01.005 22269661 PMC3312996

[B50] Goodson J. L. Kelly A. M. Kingsbury M. A. Thompson R. R. (2012b). An aggression-specific cell type in the anterior hypothalamus of finches. Proc. Natl. Acad. Sci. U.S.A. 109, 13847–13852. 10.1073/pnas.1207995109 22872869 PMC3427066

[B51] Goodson J. L. Kingsbury M. A. (2011). Nonapeptides and the evolution of social group sizes in birds. Front. Neuroanat. 5:13. 10.3389/fnana.2011.00013 21427780 PMC3049320

[B52] Goodson J. L. Kingsbury M. A. (2013). What's in a name? Considerations of homologies and nomenclature for vertebrate social behavior networks. Horm. Behav. 64, 103–112. 10.1016/j.yhbeh.2013.05.006 23722238 PMC4038951

[B53] Goodson J. L. Lindberg L. Johnson P. (2004). Effects of central vasotocin and mesotocin manipulations on social behavior in male and female zebra finches. Horm. Behav. 45, 136–143. 10.1016/j.yhbeh.2003.08.006 15019801

[B54] Goossens N. Blahser S. Oksche A. Vandesande F. Dierickx K. (1977). Immunocytochemical investigation of the hypothalamo-neurohypophysial system in birds. Cell Tissue Res. 184, 1–13. 10.1007/BF00220523 922855

[B55] Goossens N. Dierickx K. Vandesande F. (1979). Immunocytochemical localization of vasotocin and mesotocin in the hypothalamus of lacertilian reptiles. Cell Tissue Res. 200, 223–227. 10.1007/BF00236415 487396

[B56] Gould K. L. Newman S. W. Tricomi E. M. Devoogd T. J. (2001). The distribution of substance P and neuropeptide Y in four songbird species: a comparison of food-storing and non-storing birds. Brain Res. 918, 80–95. 10.1016/S0006-8993(01)02961-4 11684045

[B57] Grace M. S. Alones V. Menaker M. Foster R. G. (1996). Light perception in the vertebrate brain: an ultrastructural analysis of opsin- and vasoactive intestinal polypeptide-immunoreactive neurons in iguanid lizards. J. Comp. Neurol. 367, 575–594. 8731227 10.1002/(SICI)1096-9861(19960415)367:4<575::AID-CNE8>3.0.CO;2-1

[B58] Grant P. R. Grant B. R. (2014). 40 Years of Evolution: Darwin's Finches on Daphne Major Island. Princeton, NJ: Princeton University Press.

[B59] Gray D. A. Simon E. (1983). Mammalian and avian antidiuretic hormone: studies related to possible species variation in osmoregulatory systems. J. Comp. Physiol. 151, 241–246. 10.1007/BF00689924

[B60] Gulyas A. I. Gorcs T. J. Freund T. F. (1990). Innervation of different peptide-containing neurons in the hippocampus by GABAergic septal afferents. Neuroscience 37, 31–44. 10.1016/0306-4522(90)90189-B 1978740

[B61] Healy S. D. Krebs J. R. (1996). Food storing and the hippocampus in Paridae. Brain Behav. Evol. 47, 195–199. 10.1159/000113239 9156782

[B62] Hermes M. L. Buijs R. M. Masson-Pevet M. Pevet P. (1990). Seasonal changes in vasopressin in the brain of the garden dormouse (*Eliomys quercinus* L.). J. Comp. Neurol. 293, 340–346. 10.1002/cne.902930303 2324321

[B63] Hill W. L. Ballard S. Coyer M. J. Rowley T. (2005). The interaction of testosterone and breeding phase on the reproductive behavior and use of space of male zebra finches. Horm. Behav. 47, 452–458. 10.1016/j.yhbeh.2004.11.016 15777811

[B64] Hirunagi K. Rommel E. Oksche A. Korf H. W. (1993). Vasoactive intestinal peptide-immunoreactive cerebrospinal fluid-contacting neurons in the reptilian lateral septum/nucleus accumbens. Cell Tissue Res. 274, 79–90. 10.1007/BF00327988 8242714

[B65] Hof P. R. Dietl M. M. Charnay Y. Martin J. L. Bouras C. Palacios J. M.. (1991). Vasoactive intestinal peptide binding sites and fibers in the brain of the pigeon *Columba livia*: an autoradiographic and immunohistochemical study. J. Comp. Neurol. 305, 393–411. 10.1002/cne.903050304 1645376

[B66] Ibata Y. Okamura H. Tanaka M. Tamada Y. Hayashi S. Iijima N.. (1999). Functional morphology of the suprachiasmatic nucleus. Front. Neuroendocrinol. 20, 241–268. 10.1006/frne.1999.0180 10433864

[B67] Jarvis E. D. Yu J. Rivas M. V. Horita H. Feenders G. Whitney O.. (2013). Global view of the functional molecular organization of the avian cerebrum: mirror images and functional columns. J. Comp. Neurol. 521, 3614–3665. 10.1002/cne.23404 23818122 PMC4145244

[B68] Johansson U. S. Ekman J. Bowie R. C. K. Halvarsson P. Ohlson J. I. Price T. D.. (2013). A complete multilocus species phylogeny of the tits and chickadees (Aves: Paridae). Mol. Phylogenet. Evol. 69, 852–860. 10.1016/j.ympev.2013.06.019 23831453

[B69] Jurkevich A. Barth S. W. Aste N. Panzica G. Grossmann R. (1996). Intracerebral sex differences in the vasotocin system in birds: possible implication in behavioral and autonomic functions. Horm. Behav. 30, 673–681. 10.1006/hbeh.1996.0068 9047289

[B70] Jurkevich A. Barth S. W. Grossmann R. (1997). Sexual dimorphism of arg-vasotocin gene expressing neurons in the telencephalon and dorsal diencephalon of the domestic fowl. An immunocytochemical and in situ hybridization study. Cell Tissue Res. 287, 69–77. 10.1007/s004410050732 9011403

[B71] Jurkevich A. Grossmann R. (2003). Vasotocin and reproductive functions of the domestic chicken. Domest. Anim. Endocrinol. 25, 93–99. 10.1016/S0739-7240(03)00048-1 12963102

[B72] Kempenaers B. (1995). Polygyny in the blue tit: intra- and inter-sexual conflicts. Anim. Behav. 49, 1047–1064. 10.1006/anbe.1995.0134

[B73] Kempenaers B. Verheyen G. R. Dhondi A. A. (1997). Extrapair paternity in the blue tit (*Parus caeruleus*): female choice, male charateristics, and offspring quality. Behav. Ecol. 8, 481–492. 10.1093/beheco/8.5.481

[B74] Kimura T. Okanoya K. Wada M. (1999). Effect of testosterone on the distribution of vasotocin immunoreactivity in the brain of the zebra finch, *Taeniopygia guttata* castanotis. Life Sci. 65, 1663–1670. 10.1016/S0024-3205(99)00415-4 10573184

[B75] Kingsbury M. A. Jan N. Klatt J. D. Goodson J. L. (2015). Nesting behavior is associated with VIP expression and VIP-Fos colocalization in a network-wide manner. Horm. Behav. 69, 68–81. 10.1016/j.yhbeh.2014.12.010 25573700 PMC4359656

[B76] Kiss J. Z. Voorhuis T. A. Van Eekelen J. A. De Kloet E. R. De Wied D. (1987). Organization of vasotocin-immunoreactive cells and fibers in the canary brain. J. Comp. Neurol. 263, 347–364. 10.1002/cne.902630304 3667983

[B77] Kiyoshi K. Kondoh M. Hirunagi K. Korf H. (1998). Confocal laser scanning and electron-microscopic analyses of the relationship between VIP-like and GnRH-like-immunoreactive neurons in the lateral septal-preoptic area of the pigeon. Cell Tissue Res. 293, 39–46. 10.1007/s004410051096 9634596

[B78] Kosonsiriluk S. Sartsoongnoen N. Chaiyachet O. A. Prakobsaeng N. Songserm T. Rozenboim I.. (2008). Vasoactive intestinal peptide and its role in continuous and seasonal reproduction in birds. Gen. Comp. Endocrinol. 159, 88–97. 10.1016/j.ygcen.2008.07.024 18761341

[B79] Kuenzel W. J. Blahser S. (1991). The distribution of gonadotropin-releasing hormone (GnRH) neurons and fibers throughout the chick brain (*Gallus domesticus*). Cell Tissue Res. 264, 481–495. 10.1007/BF00319038 1868520

[B80] Kuenzel W. J. Blahser S. (1994). Vasoactive intestinal polypeptide (VIP)-containing neurons: distribution throughout the brain of the chick (*Gallus domesticus*) with focus upon the lateral septal organ. Cell Tissue Res. 275, 91–107. 10.1007/BF00305378 8118850

[B81] Kuenzel W. J. Masson M. (1988). A Stereotaxic Atlas of the Brain of the Chick (Gallus Domesticus). Baltimore, MD: Johns Hopkins University Press.

[B82] Lack D. (1968). Ecological Adaptations for Breeding in Birds. London: Methuen and Co.

[B83] Laemle L. K. Cotter J. R. (1988). Immunocytochemical localization of vasoactive intestinal polypeptide (VIP) in the brain of the little brown bat (*Myotis lucifugus*). J. Neurocytol. 17, 117–129. 10.1007/BF01735384 3047320

[B84] Lantos T. Görcs T. J. Palkovits M. (1995). Immunohistochemical mapping of neuropeptides in the premamillary region of the hypothalamus in rats. Brain Res. Rev. 20, 209–249. 10.1016/0165-0173(94)00013-F 7795657

[B85] Larsen P. J. Mikkelsen J. D. (1992). Vasoactive intestinal peptide (VIP) in magnocellular neurons of the hypothalamo-neurohypophysial system of the mink (Mustela vision) is co-localized with vasopressin or oxytocin. J. Comp. Neurol. 326, 180–192. 10.1002/cne.903260203 1479074

[B86] Leung C. H. Goode C. T. Young L. J. Maney D. L. (2009). Neural distribution of nonapeptide binding sites in two species of songbird. J. Comp. Neurol. 513, 197–208. 10.1002/cne.21947 19132730

[B87] Lorén I. Emson P. C. Fahrenkrug J. Björklund A. Alumets J. Håkanson R.. (1979). Distribution of vasoactive intestinal polypeptide in the rat and mouse brain. Neuroscience 4, 1953–1976. 10.1016/0306-4522(79)90068-X 394023

[B88] Luo Y. Peng N. Yang W. Zhang W. (1995). Studies on the distribution of vasopressin-immunoreactive neuronal perikarya and their fibers in the hypothalamus of *Tupaia belangeri*. Brain Res. 687, 191–193. 10.1016/0006-8993(95)00381-Y 7583304

[B89] Lynch K. S. Diekamp B. Ball G. F. (2012). Colocalization of immediate early genes in catecholamine cells after song exposure in female zebra finches (*Taeniopygia guttata*). Brain Behav. Evol. 79, 252–260. 10.1159/000337533 22572406 PMC3606879

[B90] Lynn S. E. (2015). Endocrine and neuroendocrine regulation of fathering behavior in birds. Horm. Behav. [Epub ahead of print]. 10.1016/j.yhbeh.2015.04.005 25896117

[B91] Macnamee M. C. Sharp P. J. Lea R. W. Sterling R. J. Harvey S. (1986). Evidence that vasoactive intestinal polypeptide is a physiological prolactin-releasing factor in the bantam hen. Gen. Comp. Endocrinol. 62, 470–478. 10.1016/0016-6480(86)90057-2 3770438

[B92] Magnone M. C. Bertolucci C. Piazza F. Foa A. (2003). Daily and circadian rhythms of neurotransmitters and related compounds in the hypothalamic suprachiasmatic nuclei of a diurnal vertebrate. Brain Res. 973, 115–121. 10.1016/S0006-8993(03)02567-8 12729960

[B93] Maley B. E. (1996). Immunohistochemical localization of neuropeptides and neurotransmitters in the nucleus solitarius. Chem. Senses 21, 367–376. 10.1093/chemse/21.3.367 8670716

[B94] Massa B. Cusimano C. A. Margagliotta B. Roberto G. (2011). Reproductive characteristics and differential response to seasonal temperatures of Blue and Great tits (*Cyanistes caeruleus* & *Parus major*) in three neighbouring mediterranean habitats. Rev. Écol. 66, 157–172. Available online at: http://documents.irevues.inist.fr/handle/2042/55874

[B95] Matthysen E. Adriaensen F. Dhondt A. A. (2011). Multiple responses to increasing spring temperatures in the breeding cycle of blue and great tits (*Cyanistes caeruleus, Parus major*). Glob. Change Biol. 17, 1–16. 10.1111/j.1365-2486.2010.02213.x

[B96] Mcgraw L. Székely T. Young L. J. (2010). Pair bonds and parental behaviour, in Social Behaviour - Genes, Ecology and Evolution, eds Székely T. Moore A. J. Komdeur J. (Cambridge: Cambridge University Press), 271–301.

[B97] Montagnese C. M. Szekely T. Gray D. Balazsa T. Zachar G. (2014). Immunoreactivity distribution of vasotocin and vasoactive intestinal peptide in brain nuclei of two songbird species with different breeding systems. Brain Behav. Evol. 83, 140–149. 10.1159/000357831 24776994

[B98] Moore F. L. Lowry C. A. (1998). Comparative neuroanatomy of vasotocin and vasopressin in amphibians and other vertebrates. Comp. Biochem. Physiol. C. Pharmacol. Toxicol. Endocrinol. 119, 251–260. 10.1016/S0742-8413(98)00014-0 9826998

[B99] Moskat C. Ban M. Szekely T. Komdeur J. Lucassen R. W. Van Boheemen L. A.. (2010). Discordancy or template-based recognition? Dissecting the cognitive basis of the rejection of foreign eggs in hosts of avian brood parasites. J. Exp. Biol. 213, 1976–1983. 10.1242/jeb.040394 20472785

[B100] Moss M. S. Basbaum A. I. (1983). The peptidergic organization of the cat periaqueductal gray. II. The distribution of immunoreactive substance P and vasoactive intestinal polypeptide. J. Neurosci. 3, 1437–1449. 6191012 10.1523/JNEUROSCI.03-07-01437.1983 PMC6564435

[B101] Nemeth J. Jakab B. Reglodi D. Lubics A. Jozsa R. Hollosy T.. (2002). Comparative distribution of VIP in the central nervous system of various species measured by a new radioimmunoassay. Regul. Pept. 109, 3–7. 10.1016/S0167-0115(02)00165-9 12409208

[B102] Norgren R. B. Jr. Silver R. (1990). Distribution of vasoactive intestinal peptide-like and neurophysin-like immunoreactive neurons and acetylcholinesterase staining in the ring dove hypothalamus with emphasis on the question of an avian suprachiasmatic nucleus. Cell Tissue Res. 259, 331–339. 10.1007/BF00318456 2337926

[B103] Obata-Tsuto H. L. Okamura H. Tsuto T. Terubayashi H. Fukui K. Yanaihara N.. (1983). Distribution of the VIP-like immunoreactive neurons in the cat central nervous system. Brain Res. Bull. 10, 653–660. 10.1016/0361-9230(83)90034-5 6347346

[B104] Otero-Garcia M. Martin-Sanchez A. Fortes-Marco L. Martinez-Ricos J. Agustin-Pavon C. Lanuza E.. (2014). Extending the socio-sexual brain: arginine-vasopressin immunoreactive circuits in the telencephalon of mice. Brain Struct. Funct. 219, 1055–1081. 10.1007/s00429-013-0553-3 23625152

[B105] Palkovits M. (1984). Distribution of neuropeptides in the central nervous system: a review of biochemical mapping studies. Prog. Neurobiol. 23, 151–189. 10.1016/0301-0082(84)90001-7 6395185

[B106] Pampus M. Schmidt K.-H. Wiltschko W. (2005). Pair bond and breeding success in Blue Tits *Parus caeruleus* and Great Tits *Parus major*. Ibis 147, 92–108. 10.1111/j.1474-919x.2004.00376.x

[B107] Panzica G. C. Aste N. Castagna C. Balthazart J. Viglietti-Panzica C. (1997). Sexual dimorphism, steroid-induced plasticity, and behavioral significance of the vasotocinergic innervation of the avian brain, in Neuroendocrinology, eds Korf H.-W. Usadel K.-H. (Berlin; Heidelberg: Springer), 127–150.

[B108] Panzica G. C. Aste N. Castagna C. Viglietti-Panzica C. Balthazart J. (2001). Steroid-induced plasticity in the sexually dimorphic vasotocinergic innervation of the avian brain: behavioral implications. Brain Res. Brain Res. Rev. 37, 178–200. 10.1016/S0165-0173(01)00118-7 11744086

[B109] Panzica G. C. Calcagni M. Ramieri G. Viglietti-Panzica C. (1988). Extrahypothalamic distribution of vasotocin-immunoreactive fibers and perikarya in the avian central nervous system. Basic Appl. Histochem. 32, 89–94. 3390126

[B110] Panzica G. C. Plumari L. García-Ojeda E. Deviche P. (1999). Central vasotocin-immunoreactive system in a male passerine bird (*Junco hyemalis*). J. Comp. Neurol. 409, 105–117. 10363714

[B111] Panzica G. C. Viglietti-Panzica C. Balthazart J. (1996). The sexually dimorphic medial preoptic nucleus of quail: a key brain area mediating steroid action on male sexual behavior. Front. Neuroendocrinol. 17, 51–125. 10.1006/frne.1996.0002 8788569

[B112] Peczely P. Kiss J. Z. (1988). Immunoreactivity to vasoactive intestinal polypeptide (VIP) and thyreotropin-releasing hormone (TRH) in hypothalamic neurons of the domesticated pigeon (*Columba livia*). Alterations following lactation and exposure to cold. Cell Tissue Res. 251, 485–494. 10.1007/BF00215858 3125979

[B113] Petko M. Ihionvien M. (1989). Distribution of substance P, vasoactive intestinal polypeptide and serotonin immunoreactive structures in the central nervous system of the lizard, *Lacerta agilis*. J. Hirnforsch. 30, 415–423. 2477439

[B114] Prakobsaeng N. Sartsoongnoen N. Kosonsiriluk S. Chaiyachet O. A. Chokchaloemwong D. Rozenboim I.. (2011). Changes in vasoactive intestinal peptide and tyrosine hydroxylase immunoreactivity in the brain of nest-deprived native Thai hen. Gen. Comp. Endocrinol. 171, 189–196. 10.1016/j.ygcen.2011.01.007 21266179

[B115] Propper C. R. Jones R. E. Lopez K. H. (1992). Distribution of arginine vasotocin in the brain of the lizard *Anolis carolinensis*. Cell Tissue Res. 267, 391–398. 10.1007/BF00302978 1600566

[B116] Puelles L. Martinez-De-La-Torre M. Paxinos G. Watson C. Martínez S. (2007). The Chick Brain in Stereotaxic Coordinates: An Atlas Featuring Neuromeric Subdivisions and Mammalian Homologies. Amsterdam: Academic Press.

[B117] Ramon Y Cajal-Agueras S. Contamina P. Parra P. Martinez-Millan L. (1986). The distribution of VIP-immunoreactive neurons in the visual cortex of adult rabbits and during postnatal development. Brain Res. 370, 333–337. 10.1016/0006-8993(86)90489-0 3518861

[B118] Reiner A. (1991). A comparison of neurotransmitter-specific and neuropeptide-specific neuronal cell types present in the dorsal cortex in turtles with those present in the isocortex in mammals: implications for the evolution of isocortex. Brain Behav. Evol. 38, 53–91. 10.1159/000114379 1683805

[B119] Reiner A. Perkel D. J. Bruce L. L. Butler A. B. Csillag A. Kuenzel W.. (2004). The avian brain nomenclature forum: terminology for a new century in comparative neuroanatomy. J. Comp. Neurol. 473, E1–E6. 10.1002/cne.20119 19626136 PMC2713747

[B120] Roberts G. W. Woodhams P. L. Bryant M. G. Crow T. J. Bloom S. R. Polak J. M. (1980). VIP in the rat brain: evidence for a major pathway linking the amygdala and hypothalamus via the stria terminalis. Histochemistry 65, 103–119. 10.1007/BF00493159 7358518

[B121] Rood B. D. De Vries G. J. (2011). Vasopressin innervation of the mouse (*Mus musculus*) brain and spinal cord. J. Comp. Neurol. 519, 2434–2474. 10.1002/cne.22635 21456024 PMC3939019

[B122] Rosen G. J. De Vries G. J. Goldman S. L. Goldman B. D. Forger N. G. (2007). Distribution of vasopressin in the brain of the eusocial naked mole-rat. J. Comp. Neurol. 500, 1093–1105. 10.1002/cne.21215 17183541

[B123] Rosen G. J. De Vries G. J. Villalba C. Weldele M. L. Place N. J. Coscia E. M.. (2006). Distribution of vasopressin in the forebrain of spotted hyenas. J. Comp. Neurol. 498, 80–92. 10.1002/cne.21032 16856162

[B124] Rosténe W. H. (1984). Neurobiological and neuroendocrine functions of the vasoactive intestinal peptide (vip). Prog. Neurobiol. 22, 103–129. 10.1016/0301-0082(84)90022-4 6382440

[B125] Seth R. Kohler A. Grossmann R. Chaturvedi C. M. (2004). Expression of hypothalamic arginine vasotocin gene in response to water deprivation and sex steroid administration in female Japanese quail. J. Exp. Biol. 207, 3025–3033. 10.1242/jeb.01118 15277557

[B126] Silveira P. F. Breno M. C. Martin Del Rio M. P. Mancera J. M. (2002). The distribution of vasotocin and mesotocin immunoreactivity in the brain of the snake, *Bothrops jararaca*. J. Chem. Neuroanat. 24, 15–26. 10.1016/S0891-0618(02)00016-9 12084408

[B127] Simerly R. B. Swanson L. W. (1987). The distribution of neurotransmitter-specific cells and fibers in the anteroventral periventricular nucleus: implications for the control of gonadotropin secretion in the rat. Brain Res. 400, 11–34. 10.1016/0006-8993(87)90649-4 2880634

[B128] Sims K. B. Hoffman D. L. Said S. I. Zimmerman E. A. (1980). Vasoactive intestinal polypeptide (VIP) in mouse and rat brain: an immunocytochemical study. Brain Res. 186, 165–183. 10.1016/0006-8993(80)90263-2 6986955

[B129] Stokes T. M. Leonard C. M. Nottebohm F. (1974). The telencephalon, diencephalon, and mesencephalon of the canary, *Serinus canaria*, in stereotaxic coordinates. J. Comp. Neurol. 156, 337–374. 10.1002/cne.901560305 4609173

[B130] Stoll C. J. Voorn P. (1985). The distribution of hypothalamic and extrahypothalamic vasotocinergic cells and fibers in the brain of a lizard, *Gekko gecko*: presence of a sex difference. J. Comp. Neurol. 239, 193–204. 10.1002/cne.902390206 4044934

[B131] Székely T. Moore A. J. Komdeur J. (2010). Social Behaviour - Genes, Ecology and Evolution. Cambridge: Cambridge University Press.

[B132] Tennyson V. M. Hou-Yu A. Nilaver G. Zimmerman E. A. (1985). Immunocytochemical studies of vasotocin and mesotocin in the hypothalamo-hypophysial system of the chicken. Cell Tissue Res. 239, 279–291. 10.1007/BF00218005 3978693

[B133] Teruyama R. Beck M. M. (2001). Double immunocytochemistry of vasoactive intestinal peptide and cGnRH-I in male quail: photoperiodic effects. Cell Tissue Res. 303, 403–414. 10.1007/s004410000313 11320656

[B134] Thepen T. Voorn P. Stoll C. J. Sluiter A. A. Pool C. W. Lohman A. H. (1987). Mesotocin and vasotocin in the brain of the lizard Gekko gecko. An immunocytochemical study. Cell Tissue Res. 250, 649–656. 10.1007/BF00218959 3690641

[B135] Valcu M. Kempenaers B. (2008). Causes and consequences of breeding dispersal and divorce in a blue tit, *Cyanistes caeruleus*, population. Anim. Behav. 75, 1949–1963. 10.1016/j.anbehav.2007.12.005

[B136] Van Eerdenburg F. J. Swaab D. F. Van Leeuwen F. W. (1992). Distribution of vasopressin and oxytocin cells and fibres in the hypothalamus of the domestic pig (*Sus scrofa*). J. Comp. Neurol. 318, 138–146. 10.1002/cne.903180203 1583158

[B137] Viglietti-Panzica C. (1986). Immunohistochemical study of the distribution of vasotocin reacting neurons in avian diencephalon. J. Hirnforsch. 27, 559–566. 3540108

[B138] Viglietti-Panzica C. Anselmetti G. C. Balthazart J. Aste N. Panzica G. C. (1992). Vasotocinergic innervation of the septal region in the Japanese quail: sexual differences and the influence of testosterone. Cell Tissue Res. 267, 261–265. 10.1007/BF00302963

[B139] Viglietti-Panzica C. Aste N. Balthazart J. Panzica G. C. (1994). Vasotocinergic innervation of sexually dimorphic medial preoptic nucleus of the male Japanese quail: influence of testosterone. Brain Res. 657, 171–184. 10.1016/0006-8993(94)90965-2 7820616

[B140] Voorhuis T. A. De Kloet E. R. (1992). Immunoreactive vasotocin in the zebra finch brain (*Taeniopygia guttata*). Brain Res. Dev. Brain Res. 69, 1–10. 10.1016/0165-3806(92)90116-E 1424081

[B141] Voorhuis T. A. Kiss J. Z. De Kloet E. R. De Wied D. (1988). Testosterone-sensitive vasotocin-immunoreactive cells and fibers in the canary brain. Brain Res. 442, 139–146. 10.1016/0006-8993(88)91441-2 3359249

[B142] Wang Z. Moody K. Newman J. D. Insel T. R. (1997). Vasopressin and oxytocin immunoreactive neurons and fibers in the forebrain of male and female common marmosets (*Callithrix jacchus*). Synapse 27, 14–25. 9268061 10.1002/(SICI)1098-2396(199709)27:1<14::AID-SYN2>3.0.CO;2-G

[B143] Wang Z. Zhou L. Hulihan T. J. Insel T. R. (1996). Immunoreactivity of central vasopressin and oxytocin pathways in microtine rodents: a quantitative comparative study. J. Comp. Neurol. 366, 726–737. 8833119 10.1002/(SICI)1096-9861(19960318)366:4<726::AID-CNE11>3.0.CO;2-D

[B144] Xie J. Kuenzel W. J. Sharp P. J. Jurkevich A. (2011). Appetitive and consummatory sexual and agonistic behaviour elicits FOS expression in aromatase and vasotocin neurones within the preoptic area and bed nucleus of the stria terminalis of male domestic chickens. J. Neuroendocrinol. 23, 232–243. 10.1111/j.1365-2826.2011.02108.x 21219483

[B145] Yamada S. Mikami S. Yanaihara N. (1982). Immunohistochemical localization of vasoactive intestinal polypeptide (VIP)-containing neurons in the hypothalamus of the Japanese quail, *Coturnix coturnix*. Cell Tissue Res. 226, 13–26. 10.1007/BF00217078 7127414

[B146] Zheng D. J. Larsson B. Phelps S. M. Ophir A. G. (2013). Female alternative mating tactics, reproductive success and nonapeptide receptor expression in the social decision-making network. Behav. Brain Res. 246, 139–147. 10.1016/j.bbr.2013.02.024 23500897 PMC3633648

